# C2 to C6 biobased carbonyl platforms for fine chemistry

**DOI:** 10.3762/bjoc.21.165

**Published:** 2025-10-15

**Authors:** Jingjing Jiang, Muhammad Noman Haider Tariq, Florence Popowycz, Yanlong Gu, Yves Queneau

**Affiliations:** 1 Université Lyon 1, INSA Lyon, CNRS, CPE Lyon, ICBMS, UMR5246, 69622 Villeurbanne, France; 2 Institute of Physical Chemistry and Industrial Catalysis, School of Chemistry and Chemical Engineering, Huazhong University of Science and Technology, 1037 Luoyu road, Wuhan 430074, China

**Keywords:** aldehydes, biobased chemistry, biomass, carbonyl, fine chemicals, ketones, multicomponent, platforms, sustainability

## Abstract

The carbonyl group is central in organic synthesis, thanks to its ability to undergo a vast range of different chemical transformations on its carbon center or at the neighboring positions. Due to the high level of oxygen content in biomass, small molecules arising from biomass often possess a carbonyl group. This is why biobased platform molecules possessing a carbonyl group, either under the form of an aldehyde, a ketone, an acid or an ester, play a dominant role in biobased chemistry. This review aims at illustrating how the chemistry of biobased carbonyl platform molecules with backbones from C_2_ to C_6_ offers opportunities to reach all kinds of chemical architectures, sometimes even complex ones benefiting from the ability of the carbonyl group to be involved in multicomponent reactions.

## Introduction

Shifting towards sustainable practices in the chemical industry relies on continued advancement of green chemistry sciences which aim to minimize the global environmental impact while maintaining the efficiency of the chemical processes [1]. Green Chemistry principles outlined by Anastas and Warner in their work “Green Chemistry: Theory and Practice (2000)” [2] notably highlight atom economy, waste minimization and the use of renewable feedstock as pillars of sustainable chemistry [3–6]. Indeed, the use of biobased feedstocks provide a promising substitute for traditional petroleum-based resources [7]. Particularly lignocellulosic materials have emerged as a key source of producing fine chemicals and fuels [8–9], relying on the advances in catalytic processes dedicated to the conversion of biomass-derived platform molecules [10–11]. Recent studies have also highlighted the potential of biobased aromatic compounds as sustainable alternatives to their fossil-derived counterparts [12–15]. Developing biobased solvents [16–17] and platform chemicals from biomass shows how renewable carbon sources can replace non-renewable chemicals in important industrial processes [18–20].

**Figure 1:**
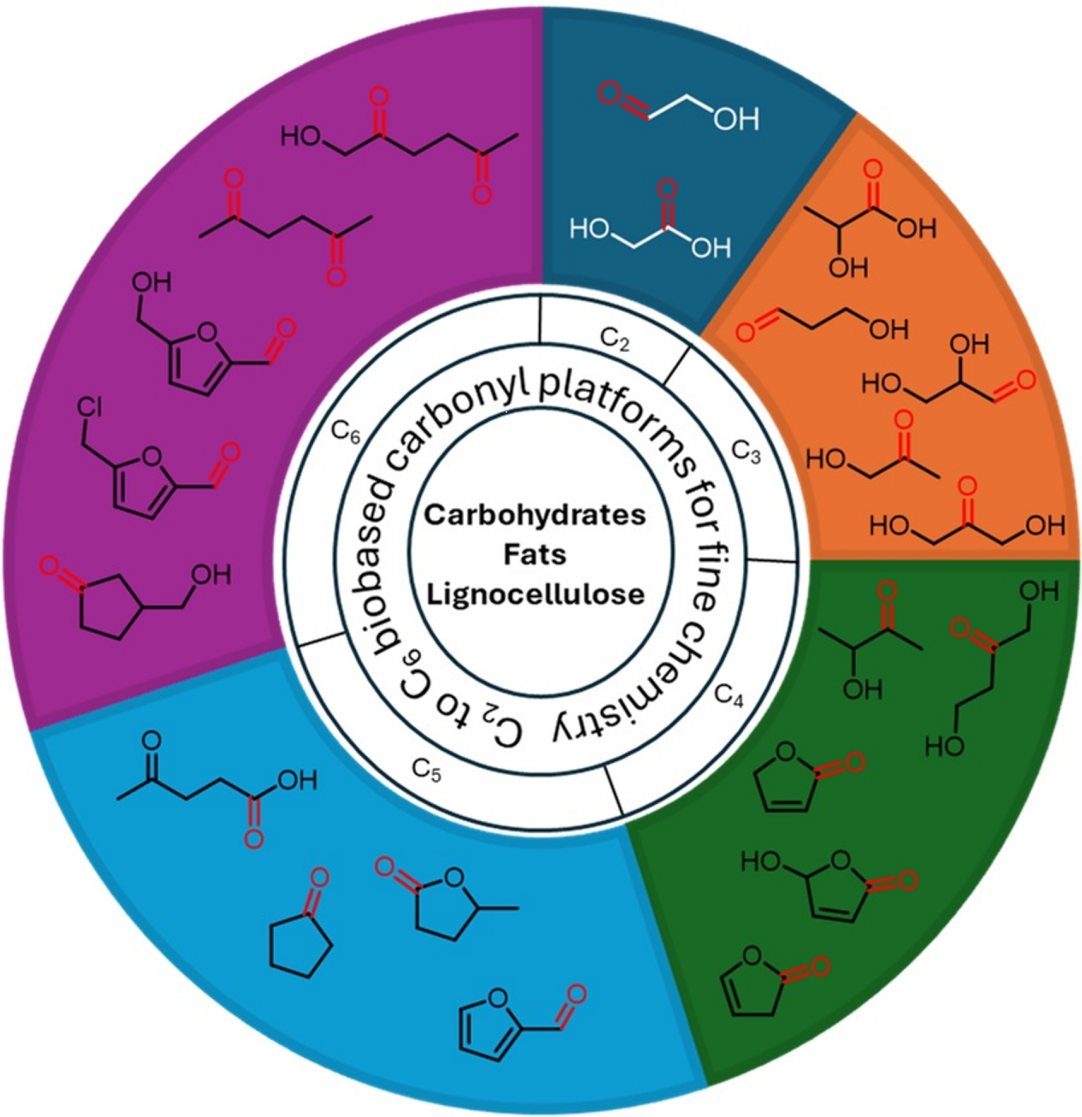
C_2_–C_6_ biobased carbonyl building blocks.

Many, if not most, biobased platform molecules contain carbonyl groups. Carbonyl compounds have unique electrophilic and nucleophilic reactivity which make them central players in organic synthesis. Therefore, synthetic chemists can base strategies on the existing reactivity of carbonyl compounds for defining novel routes converting biobased platform molecules containing carbonyl groups to prepare fine organic chemicals [21–22]. Following this approach, researchers can obtain diverse downstream products from biomass, with efforts for using clean and sustainable conditions in terms of solvents, reagents and catalysts. The topic of biomass conversion has been explored in multiple perspectives [23–27], and the purpose of the present review is to offer a specific viewpoint by focusing on the reactivity of biobased platform molecules containing carbonyl groups. This review, which does not pretend to be comprehensive, attempts to highlight the central role of carbonyl-containing biobased platform molecules notably those having a C_2_ to C_6_ backbone shown in Figure 1, and illustrating their high-value conversion methods towards fine chemicals.

## Review

### C_2_ biobased carbonyl platforms

#### Glycolaldehyde

**Scheme 1:**
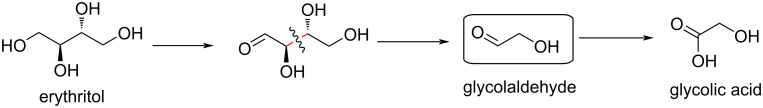
Proposed (2 + 2) route to glycolaldehyde and glycolic acid from erythritol by Cu/AC catalyst (AC = activated carbon).

Developing accesses from biomass to structures such as glycolic acid (GA) and glycolaldehyde (GCA) with high atomic economy is challenging. Hu et al. reported a new route for the synthesis of GA and GCA using activated carbon deposited Cu catalysts and different biomass-derived polyols as feedstock with exceptionally high atom utilization ≈93% and up to 90% yield (Scheme 1) [28].

**Scheme 2:**
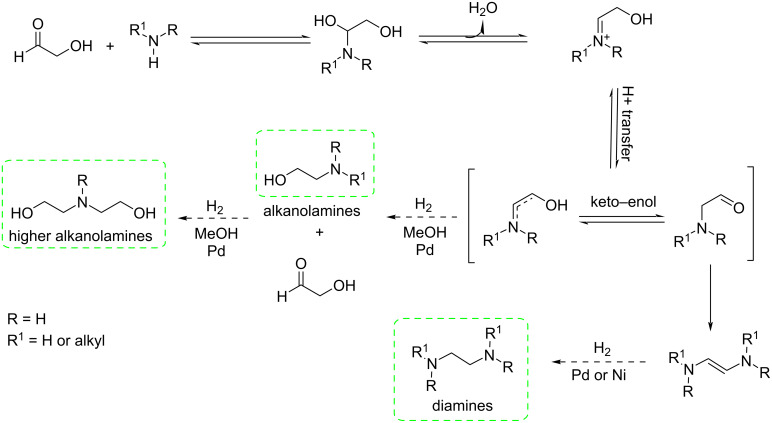
Reductive amination of GCA.

Glycolaldehyde (GCA), the smallest sugar molecule, was used by Sels et al. for the synthesis of amines by replacing the current toxic pathway based on ethylene oxide and dichloroethane [29]. Due to the high reactivity of α-hydroxycarbonyls, the main issue in this reductive amination reaction [30] was to control the cascade of consecutive and parallel reactions. The use of methanol as solvent and Pd as catalyst played a vital role for achieving a high yield. Efficient subsequent esterification and quaternarization to diesterquat fabric softeners demonstrated that high-value chemicals could be produced fully biobased from GCA as the starting platform (Scheme 2).

**Scheme 3:**
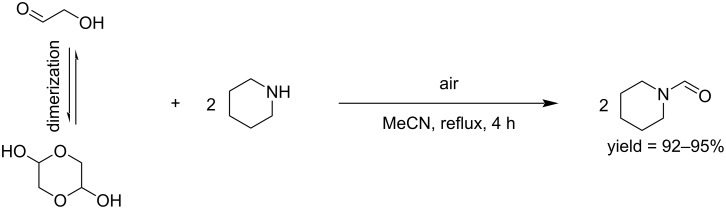
*N-*Formylation of secondary amines by reaction with GCA.

GCA can also be used as a C_1_ building block for the *N-*formylation of secondary amines to formamides under catalyst-free conditions and air as oxidant. This method was applied to different aromatic and aliphatic (both cyclic and acyclic) amines (Scheme 3) [31].

**Scheme 4:**
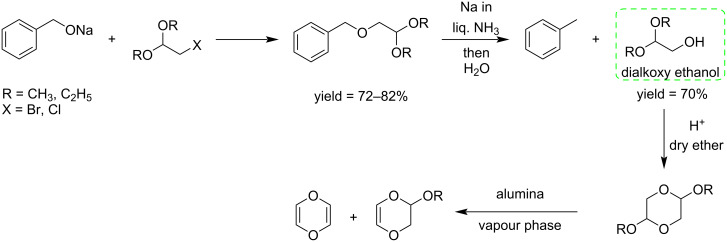
Synthesis and conversion of hydroxy acetals to cyclic acetals.

Already in 1955, Parham and Reiff reported the synthesis and conversion of a glycolaldehyde acetal (hydroxy acetal). The first step was the reaction of sodium benzyloxide with bromo acetals giving the hydroxy acetals in 75–82% yield. The intermediate ether was converted to the hydroxy acetal with sodium in liquid ammonia in good yield (70%). The hydroxy acetal then evolves towards cyclic acetals (Scheme 4) [32].

**Scheme 5:**
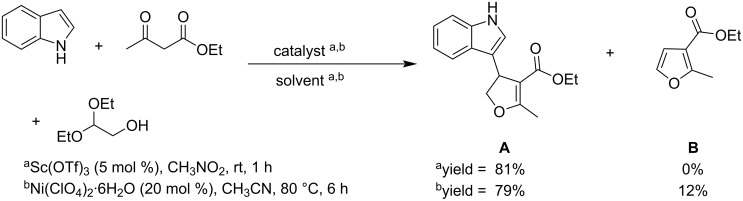
Synthesis of 3-(indol-3-yl)-2,3-dihydrofurans via three-component reaction of glycolaldehyde, indole and ethyl acetoacetate.

Exploring methods for the conversion of glycolaldehyde (GCA) to 3-(indol-3-yl)-2,3-dihydrofurans in organic solvents, Gu et al. reported a three-component reaction combining GCA, ethyl acetoacetate as 1,3-dicarbonyl component and indole. Using glycolaldehyde acetal as source of glycolaldehyde, two catalytic systems, namely Sc(OTf)_3_/nitromethane and Ni(ClO_4_)_2_·6H_2_O/acetonitrile, resulted in good yields. When using directly the aqueous solution of glycolaldehyde, these latter conditions were unproductive, whereas the deep eutectic solvent (DES) system FeCl_3_·6H_2_O/meglumine (*N*-methylglucamine) was found effective. This DES system proved to be a fully water compatible medium for the three-component conversion of glycolaldehyde, allowing the synthesis of a variety of 3-(indol-3-yl)-2,3-dihydrofurans in good yields. Interestingly, the DES (FeCl_3_·6H_2_O/meglumine) system can be recycled without significant loss of activity (Scheme 5) [33].

**Scheme 6:**
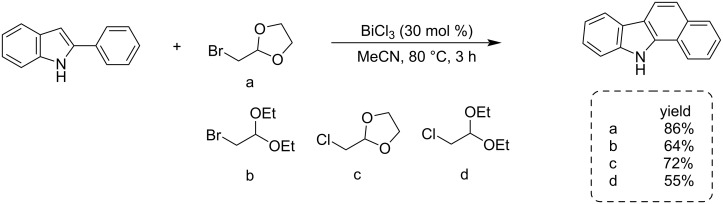
BiCl_3_-catalyzed synthesis of benzo[*a*]carbazoles from 2-arylindoles and α-bromoacetaldehyde ethylene acetal.

α-Bromoacetaldehyde derivatives (Scheme 6, structures a–d) and 2-phenylindoles were used to synthesize benzo[*a*]carbazoles. The reaction reported by the Gu research group was speeded up by bismuth trichloride (BiCl_3_). The Friedel–Crafts alkylation products were then converted into an intermediate tryptaldehyde that underwent intramolecular olefination to form the targeted product [34].

#### Glycolic acid (GA)

**Scheme 7:**
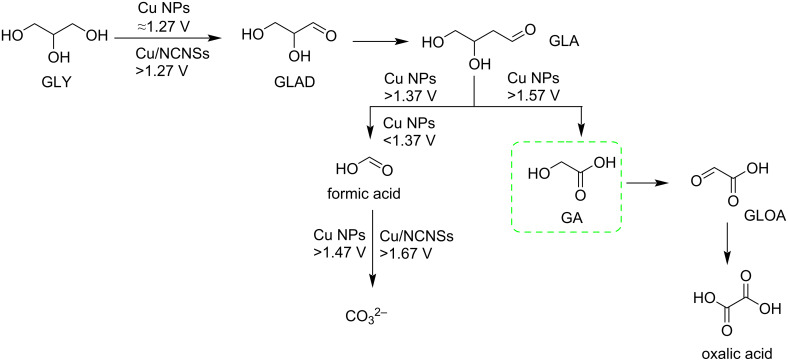
Cu/NCNSs-based conversion of glycerol to glycolic acid and other short biobased acids.

The growing impact of fossil fuel consumption has heightened the need for advancing renewable energy technologies. One promising strategy for sustainable energy development involves the electrochemical oxidation of biomass-derived feedstocks. Recent work by Shen et al. demonstrates that glycolic acid (GA), also referred to as hydroxyacetic acid, can be synthesized from glycerol (GLY) using a copper single-atom electrocatalyst supported on nitrogen-doped carbon nanosheets (Cu/NCNSs). This catalyst exhibits high activity for the oxidation of various substrates, including GLY, gluconic acid (GLU), 5-hydroxymethylfurfural (HMF), benzyl alcohol (BA), furfuryl alcohol (FA), and ethylene glycol (EG) (Scheme 7) [35].

**Scheme 8:**
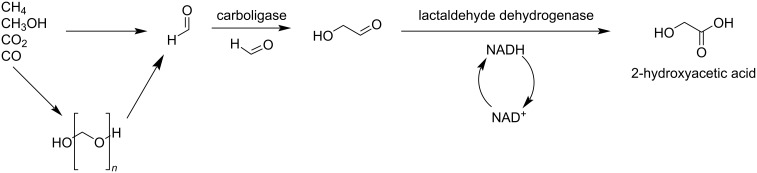
*E. coli*-based biotransformation of C_1_ source molecules (CH_4_, CO_2_ and CO) towards C_2_ glycolic acid.

**Scheme 9:**
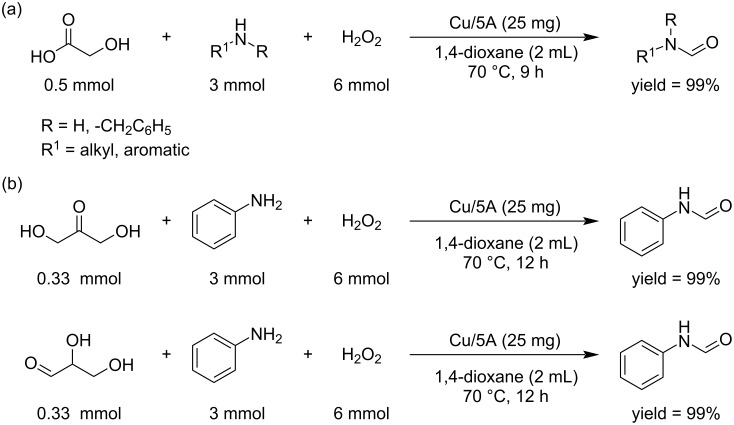
*N*-Formylation of amines with C_2_ (a) or C_3_ (b) biomass-based feedstocks.

Park et al. reported the valorization of C_1_ molecules (CH_4_, CO_2_, and CO) upgraded to the industrially important C_2_ chemical glycolic acid (GA) in a sustainable way. In the first step all the C_1_ source molecules are converted into formaldehyde. Metabolic engineering of *E. coli*-based biocatalysts allowed to avoid side reactions and promoted exclusive transformation of formaldehyde into glycolic acid. The use of *E. coli* K-12 strains as host cells and paraformaldehyde as the starting material, led to production of GA with high conversion even at rather high concentrations (Scheme 8) [36]. Using a Cu^II^-based single ion catalyst, Brückner and Shi synthesized value added formamides by treating amines with different C_2_ and C_3_ platform molecules like GA, glyceraldehyde (GLAD), and 1,3- dihydroxyacetone (DHA) (Scheme 9) [37].

### C_3_ biobased carbonyl platforms

#### Lactic acid (LA)

**Scheme 10:**
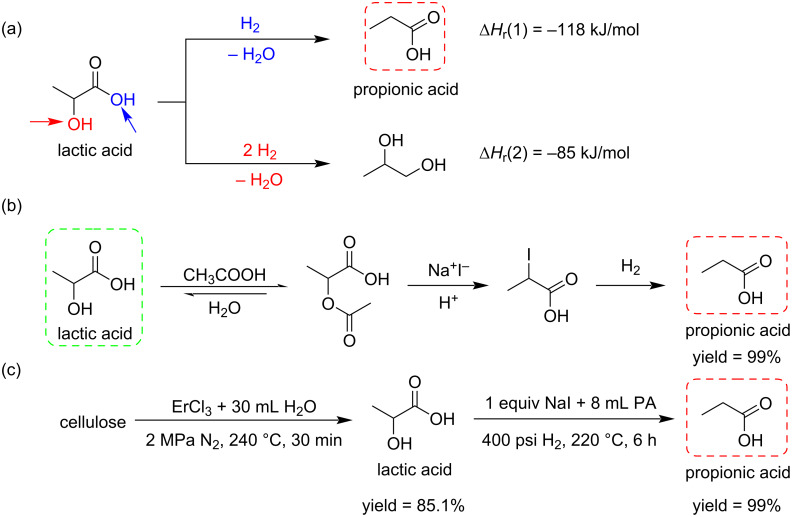
Methods for the formation of propanoic acid (PA) from lactic acid (LA).

Propanoic (or propionic) acid (PA), a valuable chemical used in the feed and food industry, is produced by petrochemical processes. Its synthesis from biobased lactic acid (LA) offers an access from renewable resources. However, this conversion is difficult due to the high activation energy required for the hydrogenation reaction removing the hydroxy group at the α-position of the carboxyl group (Scheme 10) [38]. Yang and his team reported a metal-free catalytic system for the conversion of LA to PA (Scheme 10a). The use of NaI as catalyst and PA itself as solvent allowed to simplify the product separation process, giving yields up to 99% (Scheme 10b) [39]. This strategy offers a green and efficient approach to synthesize PA from biomass resources. The synthesis of PA was also achieved in a two-step process from cellulose (Scheme 10c) [40].

**Scheme 11:**
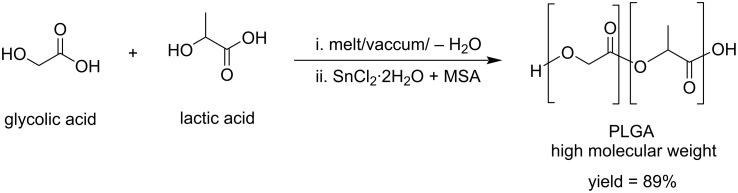
Co-polymerization of biobased lactic acid and glycolic acid via a bicatalytic process.

The use of biobased monomers is receiving tremendous attention in the field of bioplastic materials. Polylactic acid (PLA) and polyglycolic acid (PGA) are most important examples of biopolymers exhibiting interesting biodegradability properties [41]. The co-polymerization of PLA with glycolic acid was reported by Ayyoob and Kim [42]. High molecular weight poly-lactic-*co*-glycolic acid (PLGA) was obtained by the direct condensation and co-polymerization of both monomers promoted by a bicatalytic system including stannous chloride (SnCl_2_·2H_2_O) and methane sulfonic acid (MSA). PLGA films were prepared by the solvent casting technique, for which the presence of glycolic acid was found to increase their wettability (Scheme 11).

**Scheme 12:**
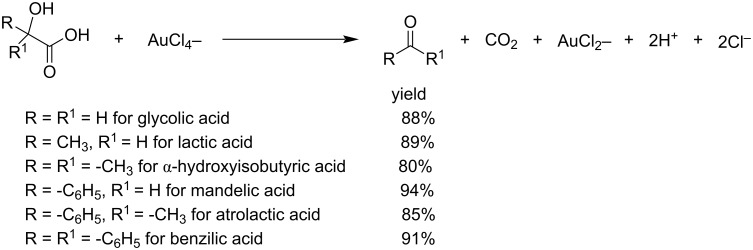
Oxidation of α-hydroxy acids by tetrachloroaurate(III) in acetic acid–sodium acetate buffer medium.

The group of Gupta et al. investigated the kinetics of the oxidation by tetrachloroaurate(III) in acetic acid–sodium acetate buffer medium of some neutralized α-hydroxy acids, including glycolic acid (GA), lactic acid (LA), α-hydroxybutyric acid (IB), mandelic acid (MA), atrolactic acid (ALA), and benzylic acid (BA). These acids were oxidized and gave formaldehyde, acetaldehyde, acetone, benzaldehyde, acetophenone, and benzophenone, respectively. By increasing pH and concentration, the rate of the reactions increased, but decreased upon increasing the amount of Cl^−^ ions (Scheme 12) [43].

**Figure 2:**
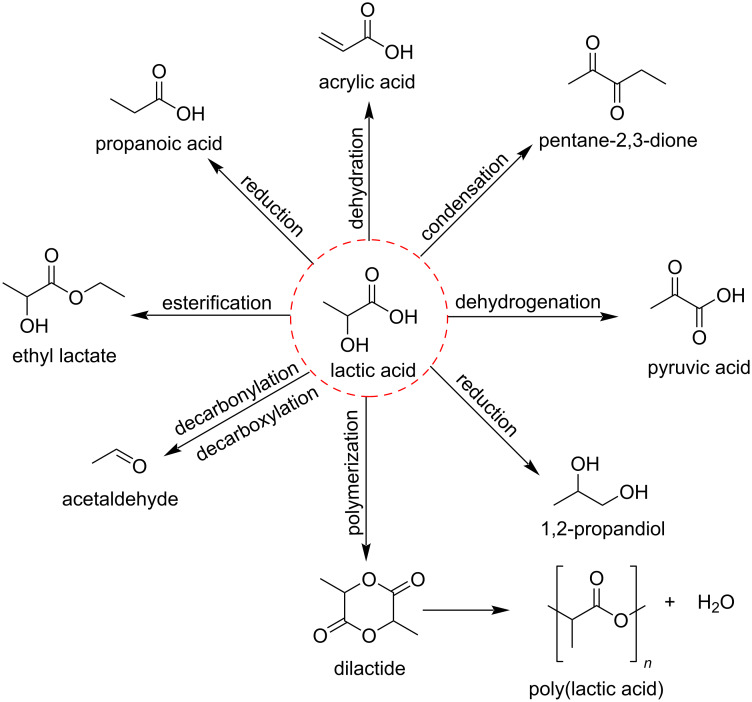
Selective catalytic pathways for the conversion of lactic acid (LA).

Many other studies have concerned lactic acid (LA) because of its low cost and versatile reactivity owing to the presence of one hydroxy group and one carboxylic group. Zhou et al. [44] summarized the selective catalytic (chemical or biological) pathways for the conversion of lactic acid towards different commodity chemicals which includes acrylic acid [45–50], pentane-2,3-dione [51–52], pyruvic acid [53–55], 1,2-propanediol [56–59], PLA [60–64], acetaldehyde [49,51], ethyl lactate [65–68] and propanoic acid (Figure 2) [49,69–70].

#### 3-Hydroxypropanal (3-HPO) and 2,3-dihydroxypropanal (2,3-HPO)

**Scheme 13:**
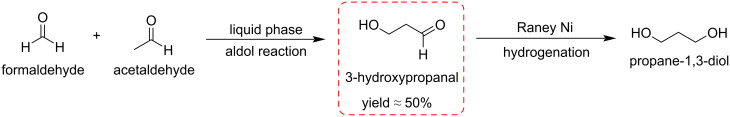
Synthesis of 1,3-PDO via cross-aldol reaction between formaldehyde and acetaldehyde to 3-hydroxypropanal followed by hydrogenation.

Xu et al. reported the cross-aldol reaction of formaldehyde and acetaldehyde for the synthesis of 3-hydroxypropanal and further reduction to 1,3-propanediol (PDO). The reaction was promoted by X-5Mg/SiO_2_ and Mg/SiO_2_ (X = Mn, Co, Ni, Fe) catalysts synthesized by the sol–gel method. The 5Mg/SiO_2_ catalyst showed the highest activity in terms of selectivity and yield because of its suitable basic and acidic density. The addition of Mn to the 5Mg/SiO_2_ (1Mn-5Mg/SiO_2_) catalyst as week acidic side increased the selectivity towards 3-HPA. According to reaction kinetics studies, the cross-aldol reaction of formaldehyde and acetaldehyde gives a higher yield of 3-HPO than the self-aldol reaction of acetaldehyde. Moreover, Raney nickel was applied successfully for the conversion of 3-HPO to 1,3-PDO with good yield and about 90% conversion rate of 3-HPO (Scheme 13) [71].

**Scheme 14:**
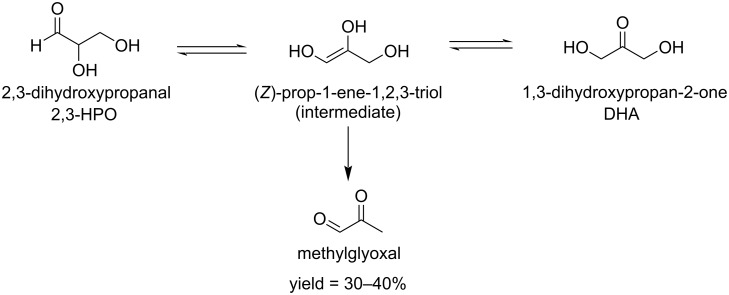
Hydrothermal conversion of 1,3-dihydroxy-2-propane and 2,3-dihydroxypropanal to methylglyoxal.

The Bobleter and Feather groups investigated the reaction mechanism of the conversion of these C_3_ compounds. The acid-catalyzed equilibrium between 1,3-dihydroxy-2-propane and 2,3-dihydroxypropanal involves an ene-triol intermediate which leads to methylglyoxal by a dehydration reaction at temperatures between 180–240 ºC in a maximum yield of about 30–40% (Scheme 14) [72–73].

#### Dihydroxyacetone (DHA)

**Scheme 15:**
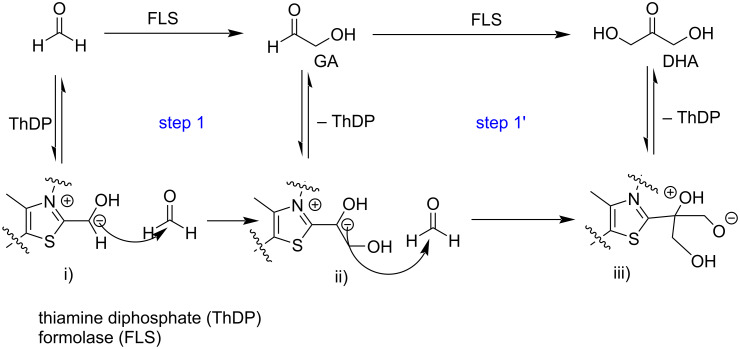
FLS-catalyzed formose reaction to synthesize GA and DHA.

The formose process is the conversion of formaldehyde to glycolaldehyde and 1,3-dihydroxy-2-propanone (dihydroxyacetone, DHA). A computational protein-directed evolution allowed to create "formolase" (FLS), an enzyme able to catalyze the formose process. Various teams investigated the kinetics of the reaction and the entire carbon uptake allowing to select the best computationally modified enzyme profile depending on the formaldehyde amount and guiding future efforts to improve this pathway (Scheme 15) [74–76].

**Scheme 16:**
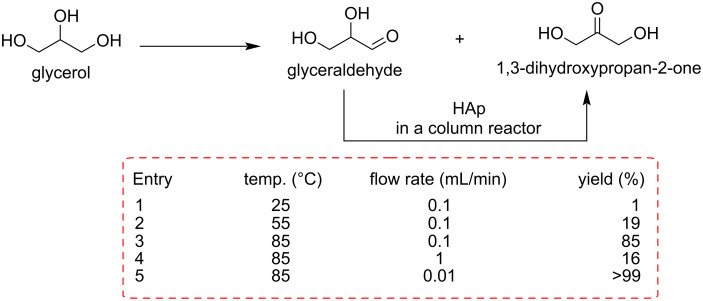
GCA and DHA oxidation products of glycerol and isomerization of GCA to DHA under flow conditions using a hydroxyapatite (Hap)-packed column.

Tang et al. recently reported the environmentally friendly production of glyceraldehyde and dihydroxyacetone from using glycerol through photocatalytic oxidation under visible light using a Cu^δ+^-decorated WO_3_ photocatalyst in the presence of hydrogen peroxide (H_2_O_2_) [77]. The presence of the photocatalyst provokes an impressive five-fold rise in the conversion rate (3.81 mmol/g) without compromising the high selectivity towards the two trioses (glyceraldehyde, 46.4%, and dihydroxyacetone, 32.9%). Okamoto and co-workers developed a new hydroxyapatite (HAp)-loaded flow system for the atom economical preparation of dihydroxyacetone (DHA) in good yield from aqueous glyceraldehyde (Scheme 16) [78].

**Scheme 17:**
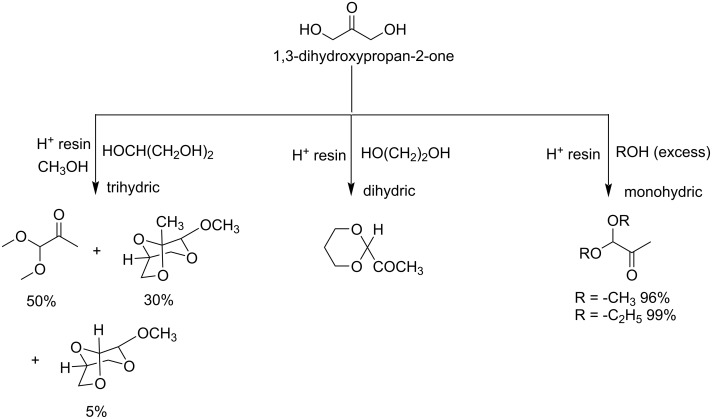
Acid-catalyzed reactions of DHA with alcohols.

Transformation of 1,3-dihydroxy-2-propanone (dihydroxyacetone, DHA) has been a very popular topic for many years, using a variety of reactions like isomerizations and dehydrations [79–81]. Keto acetals have been observed as by-products, such as in the work reported by Gupta investigating the acid-catalyzed reactions of DHA with various alcohols (Scheme 17) [82].

**Scheme 18:**
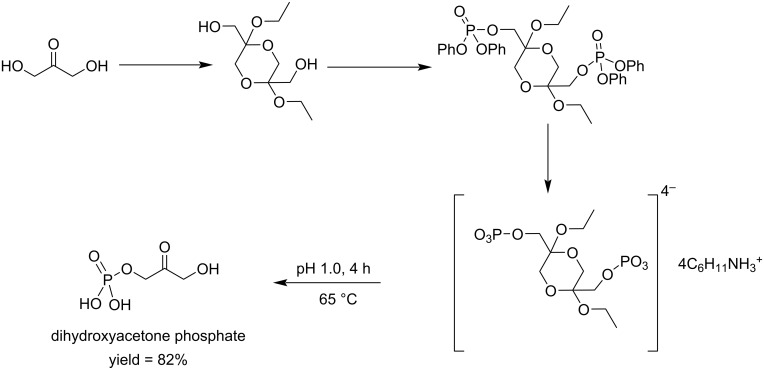
Synthesis of dihydroxyacetone phosphate from dihydroxyacetone.

Colbran and colleagues reported the conversion of DHA to dihydroxyacetone phosphate in four steps in 27% overall yield [83]. In their synthesis the cyclic acetal (2,5-diethoxy-1,4-dioxane-2,5-diyl)dimethanol or its dimethoxy analog was esterified with diphenyl phosphorochloridate in pyridine to give (2,5-diethoxy-1,4-dioxane-2,5-diyl)bis(methylene) tetraphenyl bis(phosphate). Subsequent removal of the phenyl groups through hydrogenolysis, neutralization with cyclohexylamine and treatment with ion-exchange resin led to dihydroxyacetone phosphate. This 4-step method was more efficient than the previously reported dismutation of fructose-1,6-diphosphate [84] and 3-chloro-1,3-propanediol [85] which required more than nine steps and gave only 15% yield. No remaining acetal was found in the final product (Scheme 18).

**Scheme 19:**
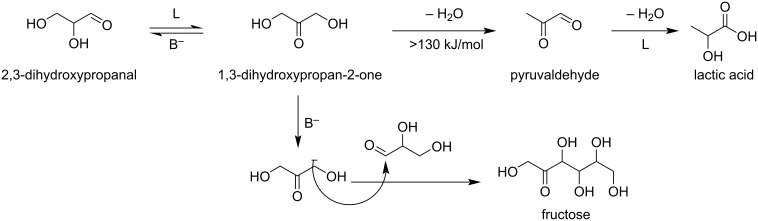
Bifunctional acid–base catalyst DHA conversion into lactic acid via pyruvaldehyde or fructose formation.

The production of lactic acid from dihydroxyacetone or its derivative pyruvaldehyde was investigated on large scale to evaluate the water tolerance of Lewis acid sites of several solid catalysts [86–92]. Essayem and co-workers reported the conversion of DHA with the help of TiO_2_-based catalysts having dual properties. Two parallel pathways are as follows: on one hand the catalyst Lewis acidic sites promote the formation of pyruvaldehyde and lactic acid (LA) by a two-step reaction, while the catalyst Lewis base sites produce fructose (Scheme 19) [93].

**Scheme 20:**
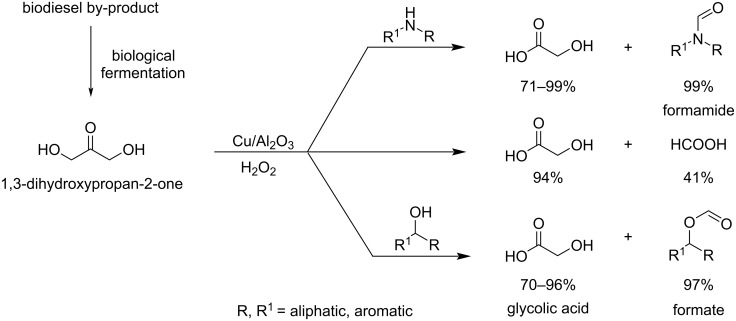
Catalytic one-pot synthesis of GA and co-synthesis of formamides and formates from DHA.

Shi et al. [94] reported a novel strategy to synthesize glycolic acid (GA), a very important building block of biodegradable polymers as already stressed in the C_2_ section. The catalytic one-pot synthesis of GA and co-synthesis of formamides and formates was achieved in particularly good yield by selective oxidation of DHA using a Cu/Al_2_O_3_ catalyst with single active Cu^II^ species at room temperature without using a base (Scheme 20).

#### 1-Hydroxypropan-2-one

**Scheme 21:**
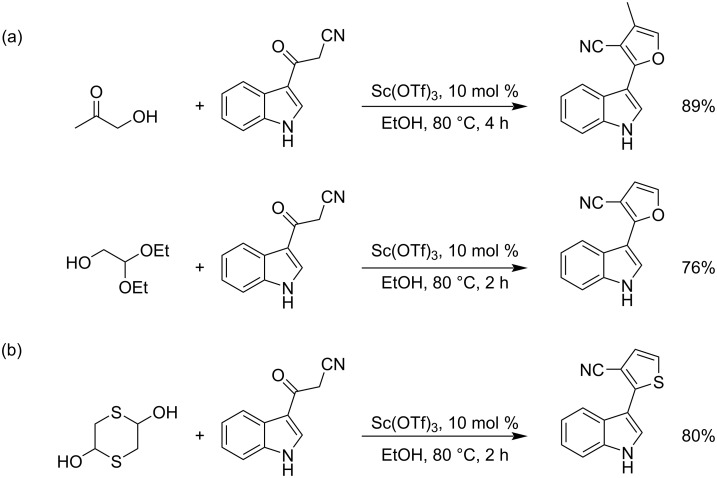
(a) Synthesis of furan derivatives and (b) synthesis of thiophene derivative by cascade [3 + 2] annulation.

Gu et al. reported the [3 + 2] cyclization reaction of 1-hydroxypropan-2-one with acylacetonitrile towards furan derivatives using a Sc(OTf)_3_ catalyst. C_2_-based bifunctional acetals, such as glycolaldehyde diethyl acetal, α-bromoacetaldehyde acetal, and 1,4-dithiane-2,5-diol also take part in the reaction as counterpart reagents. The well-known fungicide fenfuram was prepared using this process. Antifungal activity assays against four general plant fungi demonstrated that several of the synthesized new furan products exhibited also broad and strong biological activities (Scheme 21) [95].

**Scheme 22:**
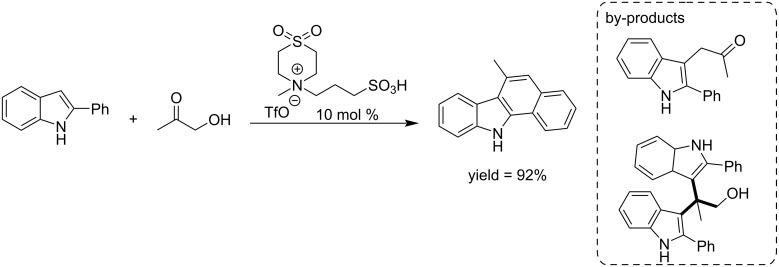
Brønsted acidic ionic liquid catalyzed synthesis of benzo[*a*]carbazole from renewable acetol and 2-phenylindoles.

A metal-free efficient strategy was developed by the Gu group for the synthesis of benzo[*a*]carbazole (a very important scaffold in the pharmaceutical industry) from the commercially available 2-phenylindole and bio-renewable 1-hydroxypropan-2-one (acetol) using a sulfone-containing Brønsted acidic medium as catalyst. The process was applied to a number of substituted 2-phenylindoles and hydroxyketones. The catalyst could be recovered and reused five times without decreasing its selectivity and activity. Mechanistically, the reaction involves a Heyns-type rearrangement and subsequent intramolecular olefination (Scheme 22) [96].

**Scheme 23:**
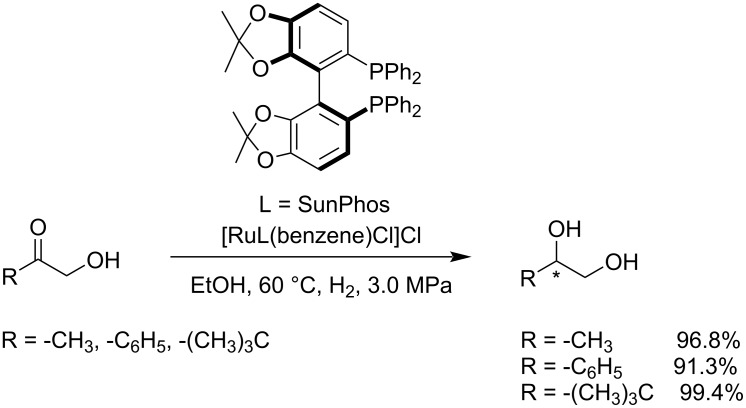
Asymmetric hydrogenation of α-hydroxy ketones to 1,2-diols.

Arandia et al. reported the application of the aqueous-phase reforming (APR) technology for the production of light hydrocarbons and hydrogen in a single step from aqueous organic phases [97] containing 1-hydroxypropan-2-one (acetol), ethanol, benzene-1,2-diol (catechol), acetic acid or mixtures thereof. The process was conducted over various Ni-based catalysts including Ni/CeO_2_-γAl_2_O_3_, spinal NiAl_2_O_4_ and Ni/La_2_O_3_-*α*Al_2_O_3_, at 230 °C and 3.2 MPa. Using a chiral catalyst composed of [RuCl_2_(benzene)]_2_ and SunPhos, an effective asymmetric hydrogenation of α-hydroxy ketones was reported, yielding chiral terminal 1,2-diols in up to 99% ee. This Ru-catalyzed asymmetric hydrogenation process of α-hydroxy ketones opens up a new pathway for the production of chiral terminal 1,2-diols (Scheme 23) [98].

**Scheme 24:**
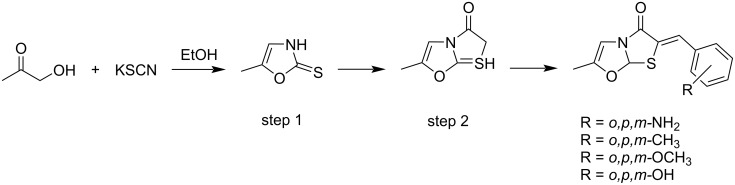
Synthesis of novel 6-(substituted benzylidene)-2-methylthiazolo [2,3-*b*]oxazol-5(6*H*)-one from 1-hydroxypropan-2-one.

Kini and Mathews reported the synthesis of novel oxazole derivatives such as 6-(substituted benzylidene)-2-methylthiazolo[2,3-*b*]oxazol-5(6*H*)-one by reacting 1-hydroxypropan-2-one and KSCN in ethanol for further evaluation of their anti-cancerous activity by MTT assays (Scheme 24) [99].

**Scheme 25:**
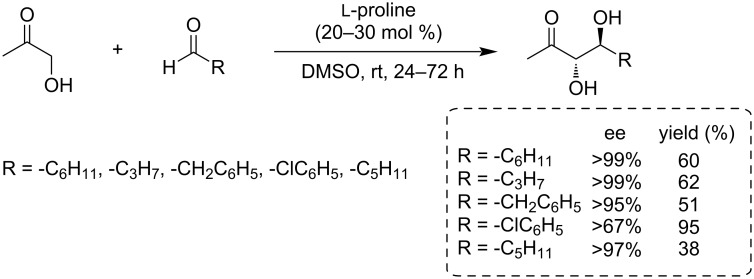
ʟ-Proline-catalyzed synthesis of *anti*-diols from hydroxyacetone and aldehydes.

**Scheme 26:**
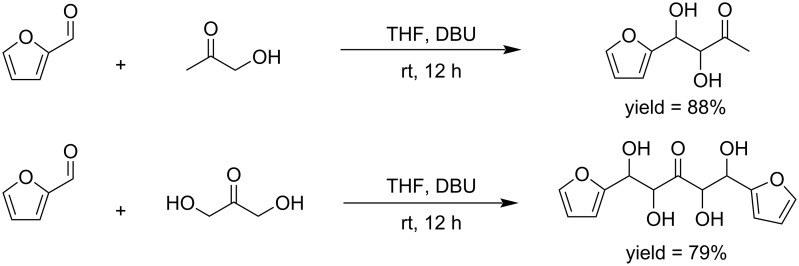
C–C-bond-formation reactions of a biomass-based feedstock aromatic aldehyde (C_5_) and hydroxyacetone (C_3_).

Notz and List reported the synthesis of *anti*-diols in good yield from 1-hydroxypropan-2-one and different (aromatic and aliphatic) substituted aldehydes using a new strategy using ʟ-proline as a catalyst (Scheme 25) [100]. The C–C-bond formation between biomass-based feedstock by aldol condensation reactions of furfural with 1-hydroxyacetone has been reported by Subrahmanyam and co-workers (Scheme 26) [101].

### C_4_ biobased carbonyl platforms

#### Acetoin

**Scheme 27:**
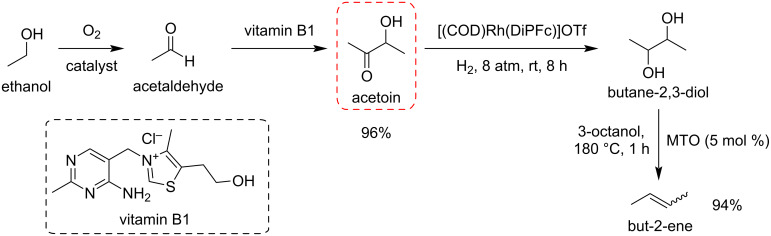
Ethanol upgrading to C_4_ bulk chemicals via the thiamine (VB1)-catalyzed acetoin condensation.

Acetoin is a very important C_4_ biomass-based molecule and widely used building block in the food industry as flavoring compound. It can be synthesized from diacetyl by reduction using enzymes such as *Aerobacter aerogenes* [102] or thiamine diphosphate-dependent lyase (ThdP-lyase) [103]. The biotechnological production of chiral acetoin was reported by Meng and co-workers [104]. Zhang and his team [105] reported the synthesis of bulk C_4_ chemicals from bioethanol via intermediate formation of acetoin in the presence of simple, environmentally benign, cheap and readily available natural vitamin B1 salts as the key step. Acetoin was further converted to C_4_ bulk chemicals such as 2,3-butanediol and butene (Scheme 27).

#### 1,4-Dihydroxybutan-2-one

**Scheme 28:**
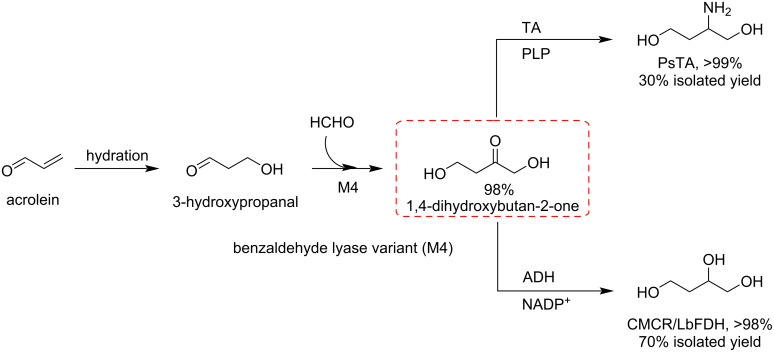
One-pot sequential chemoenzymatic synthesis of 2-aminobutane-1,4-diol and 1,2,4-butanetriol via 1,4-dihydroxybutan-2-one.

1,4-Dihydroxybutan-2-one is a key intermediate for the synthesis of different polyols such as 2-aminobutane-1,4-diol. Ma and co-workers synthesized 1,4-dihydroxybutan-2-one by a benzaldehyde lyase-catalyzed reaction of formaldehyde with 3-hydroxypropanal [106]. Another report depicts a new M4-catalyzed reaction between acrolein and formaldehyde for the synthesis of 1,4-dihydroxybutan-2-one, which was further converted into 1,2,4-butanetriol and 2-aminobutane-1,4-diol (Scheme 28) [107].

**Scheme 29:**
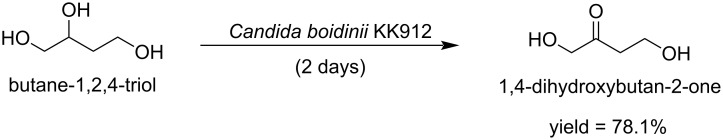
Synthesis of 1,4-dihydroxybutan-2-one by microbial transformation.

Matsumura et al. [108] reported the biocatalytic conversion of butane-1,2,4-triol into 1,4-dihydroxybutan-2-one. This work dealt with the conversion of *sec*-hydroxy groups to carbonyl derivatives by methanol yeast, *Candida boidinii* KK912, a cheaper and simpler process compared to other methods. The product is a kind of tetrose with biological and industrial relevance (Scheme 29).

**Scheme 30:**
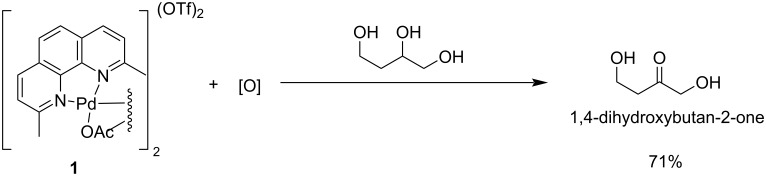
Conversion of polyols by [neocuproine)Pd(OAc)]_2_(OTf)_2_] to α-hydroxy ketones.

**Scheme 31:**
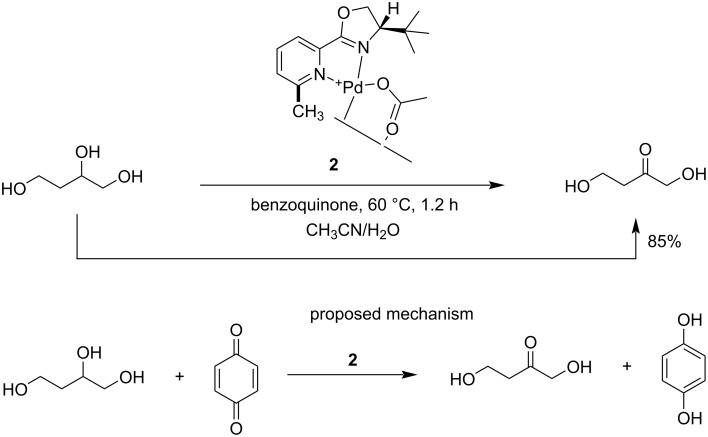
Chemoselective oxidation of alcohols with chiral palladium-based catalyst **2**.

Waymouth et al. [109] reported the regio- and chemoselective oxidation of vicinal polyols using [2,9-dimethyl-1,10-phenanthroline = neocuproine)Pd(OAc)]_2_(OTf)_2_] (**1**) as catalyst. The formation of α-hydroxyketones occurs very rapidly under mild conditions. The catalyst **1** oxidizes the vicinal diols to the corresponding hydroxy ketones faster than other alcohols. Primary alcohols and 1,5-diols are oxidized to yield secondary alcohols and cyclic lactones, respectively (Scheme 30). A high chemo- and regioselectivity was observed for the oxidation of 1,2-diols, triols and tetraols using the chiral palladium-based catalyst **2** bearing a pyridinyl oxazoline (pyOX) ligand (Scheme 31) [110].

#### 5-Hydroxy-2(5*H*)-furanone (HFO)

**Scheme 32:**
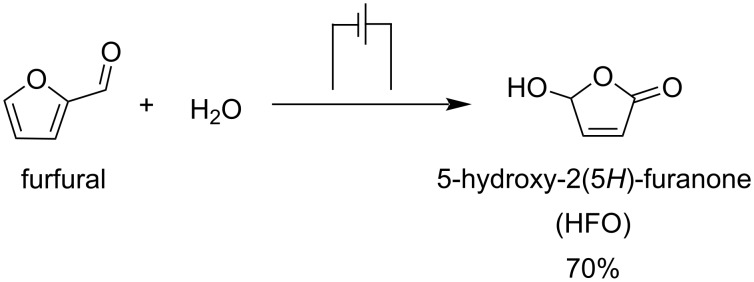
Electrochemical transformation of furfural to 5-hydroxy-2(5*H*)-furanone (HFO).

5-Hydroxy-2(5*H*)-furanone (HFO) is an interesting C_4_ platform which is formed from furfural by oxidation. Han and co-workers developed an electrocatalytic strategy for the synthesis of HFO from furfural [111]. The very important and key bioactive compound HFO was formed by using water as an oxygen source and chalcogenides such as CuS, ZnS, or PbS as electrocatalysts. CuS nanosheets gave the best performance, with high selectivity towards HFO (83%) and high conversion of furfural (70%) (Scheme 32).

**Scheme 33:**
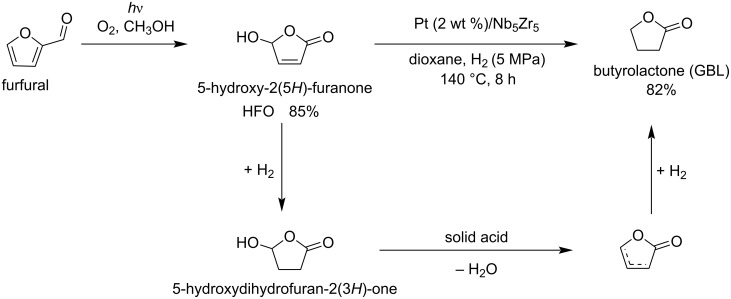
Selective hydrodeoxygenation of HFO and oxidation to γ-butyrolactone (GBL).

Yuan et al. [112] reported the synthesis of γ-butyrolactone (GBL) by the hydrodeoxygenation of HFO prepared by photocatalytic conversion of furfural. HFO is converted to GBL by two pathways: the first one is dehydration over mesoporous solid acids and the second one is a metal-catalyzed hydrogenation. HFO was obtained in 85% yield and its conversion rate to GBL was about 97%. Different bifunctional Pt-based solid acids were used. An overall GBL yield of 82.7% from furfural was obtained (Scheme 33).

**Scheme 34:**
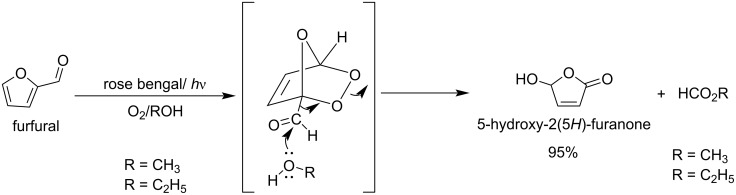
Photosensitized oxygenation of furan towards HFO via ozonide intermediates.

Gollnick and Griesbeck reported the tetraphenylporphin-photosensitized oxygenations of furan and derivatives in non-polar aprotic solvents, yielding the corresponding monomeric unsaturated secondary ozonides through a (4 + 2) cycloaddition of singlet oxygen onto the diene linkage of the furan ring. The attack of a hydroxy group on the carbonyl group of the ozonide triggers the formation of HFO in high yields (>90%) (Scheme 34) [113–115].

**Scheme 35:**
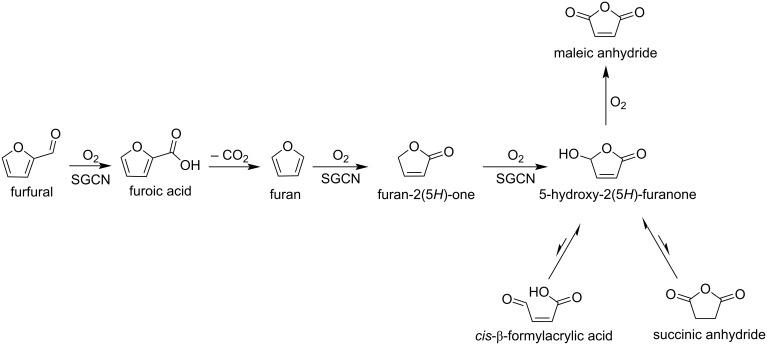
Conversion of furfural to HFO and MAN by using mesoporous carbon nitride (SGCN) as photocatalyst.

Kailasam and co-workers reported a heterogeneous photocatalytic oxidation of furfural towards HFO and maleic anhydride (MAN) [116]. This conversion is performed under simulated solar light and molecular O_2_ as the oxidant, and mesoporous carbon nitride (SGCN) as the photocatalyst. The latter showed excellent photoconversion (>95%) of furfural with 33% and 42% selectivity of HFO and MAN, respectively (Scheme 35). Xiao et al. developed this method and suggested that the Fe_2_B_2_ can be used as electrocatalyst for the production of furanoic acid and HFO with potentials of −0.15 V and −0.93 V, respectively [117].

**Scheme 36:**
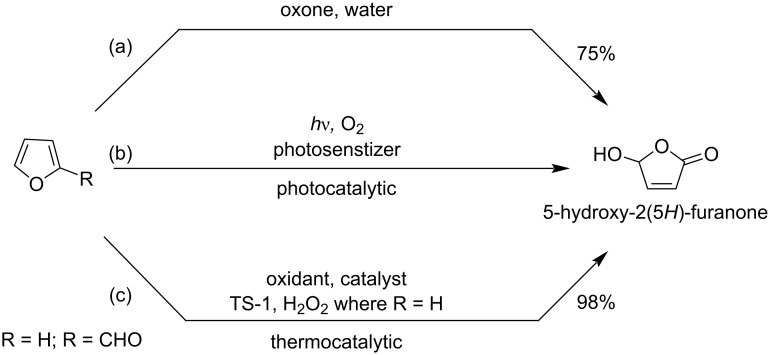
Synthesis of HFO from furan derivatives.

Chavan et al. reported a simple and efficient method for the synthesis of HFO from furan by using oxone as oxidant (Scheme 36a) [118]. Before this study Kumar et al. also synthesized HFO from furan by thermocatalytic oxidation (Scheme 36b) [119]. Salomon and co-workers reported the conversion of 2-substituted furans to HFO using NaClO_2_ as oxidizing agent (Scheme 36c) [120].

**Scheme 37:**
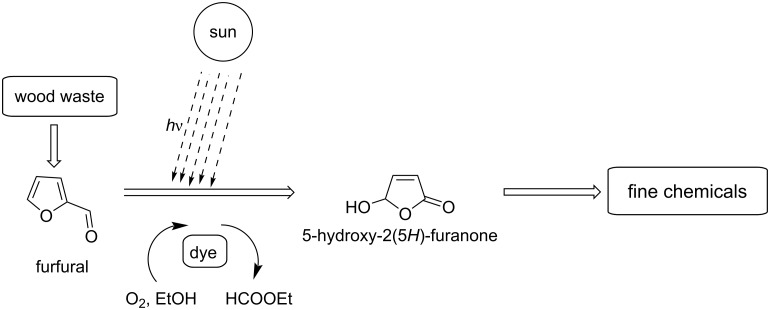
Photooxidation of furfural to 5-hydroxy-2(5*H*)-furanone (HFO).

The conversion of furfural to HFO by a dye-sensitized photooxidation reaction with oxygen in alcoholic medium has also been reported (Scheme 37) [121–122].

**Scheme 38:**
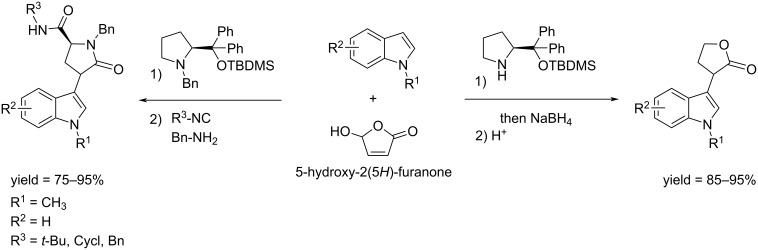
Synthesis of Friedel–Crafts indole adduct from HFO.

Riguet synthesized γ-lactams through a Ugi 4-center 3-component reaction (U-4C-3CR) protocol. HFO was used as the electrophile in the Friedel–Crafts (FC) alkylation reactions of indole catalyzed by diphenylprolinol silyl ether. The high reactivity of HFO permitted that a limited loading of catalyst could be employed. The reduction of the Friedel–Crafts adduct gave the targeted indoyl lactones with good yield (Scheme 38) [123].

**Scheme 39:**
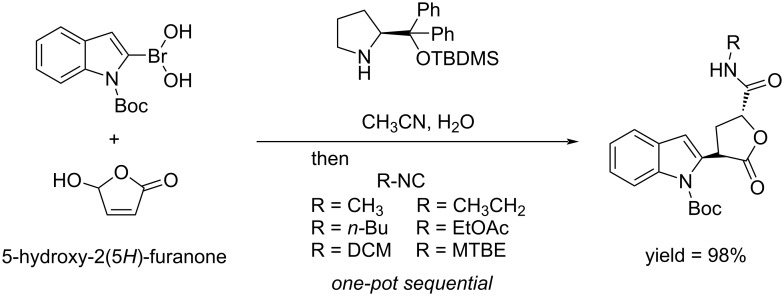
Conversion of HFO to α,γ-substituted chiral γ-lactones.

Bos and Riguet reported the one pot synthesis of α,γ-substituted chiral γ-lactones from HFO. The reaction involves the enantioselective organocatalyzed transfer of boronic acid to HFO followed by an intramolecular diastereoselective Passerini-type reaction. Varying the boronic acid enabled to reach high yields (Scheme 39) [124].

**Scheme 40:**
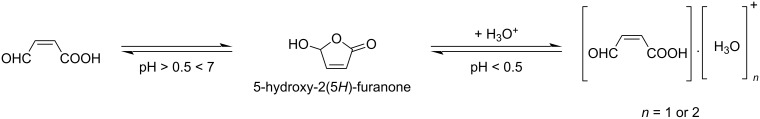
Tautomeric transformation of HFO to formylacrylic acid.

**Scheme 41:**
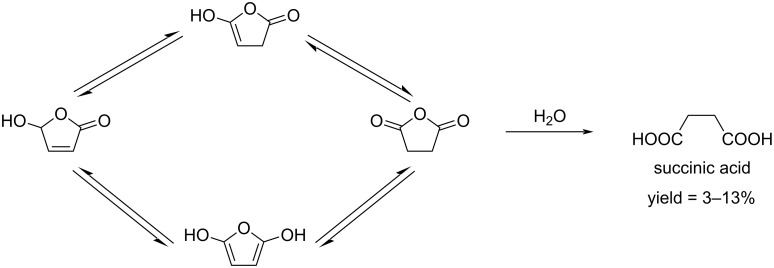
Hydrolysis of HFO to succinic acid in aqueous solution.

The tautomeric transformation of HFO to *cis*-β-formylacrylic acid was reported by Strizhov et al. [125]. The liable equilibrium transformation requires precisely pH-controlled conditions. In neutral medium, the furanone is in the form of its cis tautomer, while upon a slight increase in the pH, its cyclic original tautomer undergoes a ring opening with the formation of protonated forms (Scheme 40). HFO can be hydrolyzed to succinic acid, however, it is a slow process in aqueous solution. In NaOH solution at pH 9–10, a complete conversion of HFO to succinic acid is rapidly achieved (Scheme 41) [126].

**Scheme 42:**
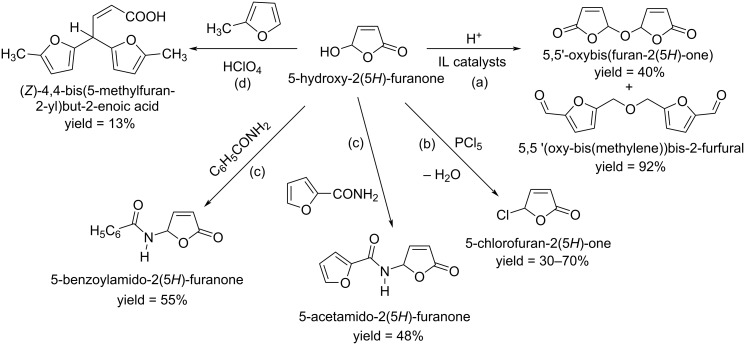
Substitution and condensation reactions of 5-hydroxy-2(5*H*)-furanone (HFO).

Substitution and condensation reactions of HFO (crude from furfural oxidation without isolation) produce 5,5'-oxybis(furan-2(5*H*)-one) and 5,5 '-(oxybis(methylene))bis-2-furfural [127–129] (Scheme 42, path a). The antimicrobial agent 5-chloro-2(5*H*)-furanone was synthesized by reaction of HFO with thionyl chloride in 30% yield [130], or with phosphorus chloride in 70% yield (Scheme 42, path b) [131]. 5-Acetamido-2(5*Н*)-furanone is obtained in 20–40% yield when acetamide reacts with 5-bromo- or 5-ethoxy-2(5*H*)-furanone [132–135], while 5-acetamido-2(5*Н*)-furanone and 5-benzoylamido-2(5*H*)-furanone were obtained in 48% and 55% yield, respectively, by reaction of HFO with benzoic acid amide or furan-2-carboxamide in 1:1 molar ratio at 70 °C for 4–8 h (Scheme 42, path c) [132]. The reaction of HFO with 2-methylfuran in a 1:2.2 molar ratio in diethyl ether solution at room temperature in the presence of catalytic amounts of perchloric acid for 5 hours led to the formation of *gem*-bis-4,4-(5-methyl-2-furyl)-2-butenoic acid in 13% yield (Scheme 42, path d) [131].

**Scheme 43:**
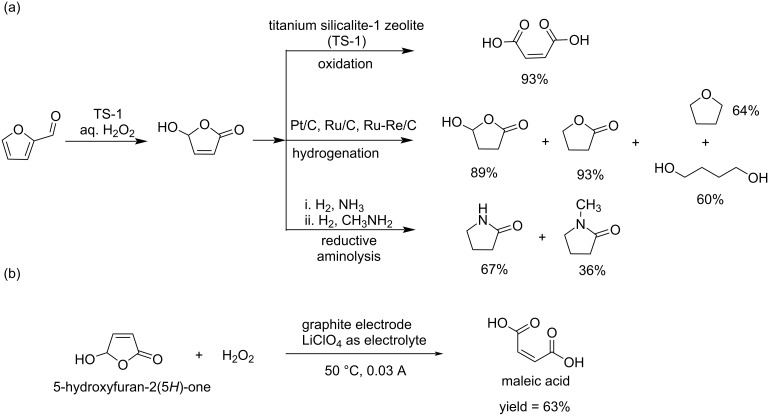
(a) Conversion of HFO towards valuable C_4_ chemicals and (b) anodic oxidation of 5-hydroxy-2(5*H*)-furanone to maleic acid.

Industrial chemicals with four carbon structures like pyrrolidines, 1,4-butanediol, maleic acid, and butyrolactone are often petroleum-based. However, developing sustainable biomass-derived routes for these compounds remains challenging. In a significant advancement, Shrotri et al. demonstrated an efficient catalytic process for synthesizing C_4_ chemicals from biomass-derived hydroxymethylfurfural (HMF) using a TS-1 catalyst under mild reaction conditions [136]. Their work showed that HFO, when processed at moderate temperatures (100–150 °C), could be selectively converted into valuable C_4_ products with remarkable yields: maleic acid and γ-butyrolactone were both obtained in 93% yield, while 1,4-butanediol, tetrahydrofuran, and 2-pyrrolidone were produced in 60%, 64%, and 67% yields, respectively (Scheme 43a). In this field, Badovskaya and co-workers chose the anodic oxidation of HFO with H_2_O_2_ using electrodes made of graphite and LiClO_4_ as supporting electrolyte at 50 °C and 0.03 A current, providing maleic acid in 63% yield (Scheme 43b) [129].

**Figure 3:**
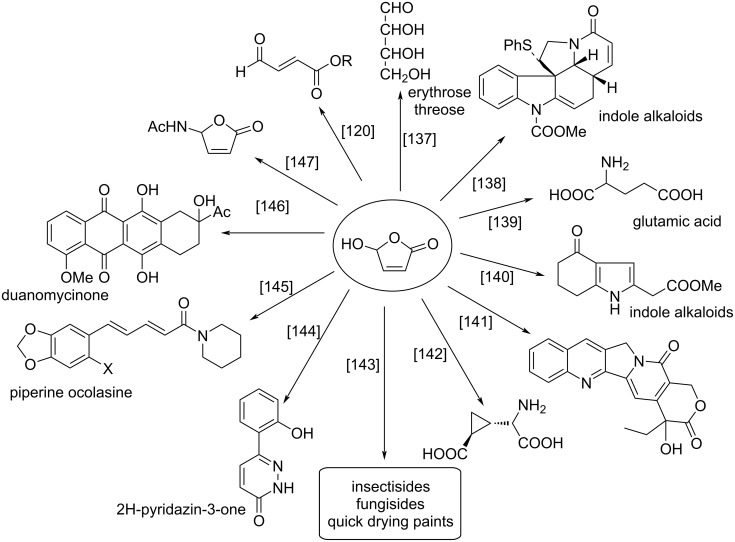
Conversion of HFO towards other natural and synthetic substances.

There are other important conversions of HFO serving as a C_4_ building block applied in the synthesis of many different substances. These are summarized in Figure 3 below [120,137–147].

#### Other furanones

**Scheme 44:**
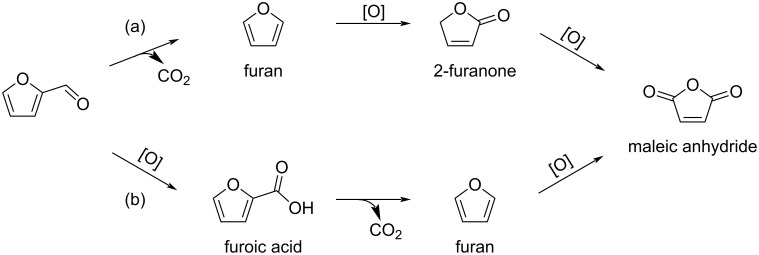
Conversion of furfural to maleic anhydride (reaction a: VO_x_/Al_2_O_3_; reaction b: VPO).

Several other furanones are key intermediates towards important chemicals. For example, the direct oxidation of furfural under aerobic conditions provides maleic anhydride via the successive oxidative decarboxylation of furfural to furan, the oxidation of furan to furanone, and the final oxidation of furanone to maleic anhydride in the presence of VO_x_/Al_2_O_3_ catalysts (Scheme 44, route a) [148]. When using a vanadium phosphorus oxide catalyst (VPO), furoic acid was also found to be a key intermediate (Scheme 44, route b) [149]. A photocatalytic access to succinic anhydride from furoic acid using catalytic porphyrin H_2_TPP in the presence of O_2_ and light was also reported [150].

**Scheme 45:**
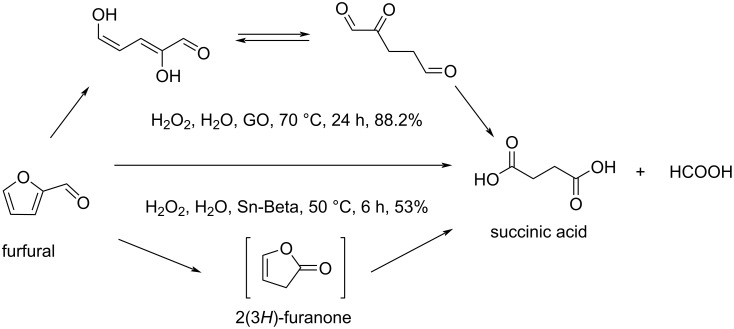
Conversion of furfural into succinic acid.

Yang and Lv reported the oxidation of furfural to succinic acid in 88% yield under mild conditions using modified hydrophilic acidic metal-free graphene oxide (GO) as solid acid catalyst and H_2_O_2_ as an oxidant [151]. The suitable acidity of the SO_3_H group on the graphene oxide support is crucial for the selectivity of the oxidation. The use of the Lewis acidic Sn-Beta was also reported for this reaction providing succinic acid in 53% yield. Sn-Beta accelerated the Baeyer–Villiger oxidation of furfural to the 2(3*H*)-furanone intermediate by activating furfural (Scheme 45) [152]. Two methods, one using CO under palladium catalysis, and one using electrocatalysis and CO_2_ have been reported to produce furandicarboxylic acid (FDCA) from bromofuroic acid [153–154].

**Scheme 46:**
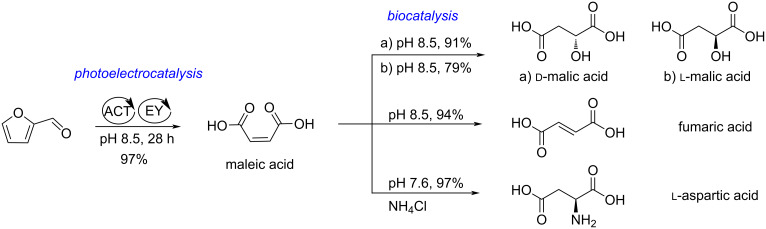
Electro‑, photo‑, and biocatalysis for one-pot selective conversions of furfural into C_4_ chemicals.

A combined electro-, photo-, and biocatalytic approach to several different C_4_ chemicals from furfural, including maleic acid, ᴅ-malic acid, ʟ-malic acid, fumaric acid, and ʟ-aspartic acid has been reported (Scheme 46). Maleic acid was produced in 97% yield through the electrochemical oxidation of furfural with 4-acetamido-2,2,6,6-tetramethylpiperidine-*N*-oxyl (ACT) and the photooxygenation with eosin Y (EY). Maleic acid was then selectively converted to ᴅ-malic acid, ʟ-malic acid, fumaric acid and ʟ-aspartic acid by biocatalysis in 91%, 79%, 94%, 97% yield, respectively, in the presence of enzymes such as maleate hydratase, maleate *cis–trans* isomerase, fumarase, or ʟ-aspartase [155].

### C_5_ biobased carbonyl platforms

#### Furfural

**Scheme 47:**
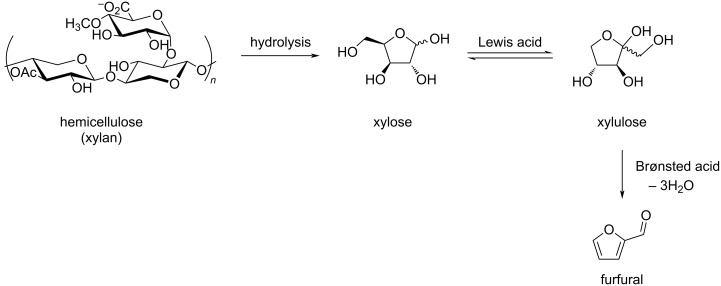
Production route of furfural from hemicellulose.

Furfural is a versatile biobased C_5_ platform molecule which plays an important role in the biorefining industry. It was first isolated in 1832 by the German chemist Johann Wolfgang Döbereiner, and its structure was determined in 1901 by German chemist Carl Harries. In 1922, the Quaker Oats Company produced furfural on a large scale using oat hulls. Since, many efforts have addressed several issues of the original industrial process, such as low yield, high energy consumption, difficult treatment of acidic aqueous waste and other wastes. Furfural is now produced on an industrial scale in batch or continuous reactors from lignocellulosic resources such as bagasse, corncobs, stalks, switchgrass and hardwoods [156–162]^,^ thanks to preliminary selective dissolution and depolymerization of hemicellulose into pentose sugars. Deep eutectic solvents were recently shown to allow fractionation of lignin and production of furfural from lignocelluloses [163]. The hydrolysis and dehydration process of hemicellulose can be accomplished in water or in aqueous-based biphasic systems in the presence of mineral acids (such as H_2_SO_4_, HCl) and organic acids (such as HCOOH, CH_3_SO_3_H). The acid catalysts used for furfural production range from homogeneous catalysts such as mineral acids, organic acids, metal salts (with high availability and low costs) to heterogeneous ones such as zeolites, metal oxides, sulfonated polymers, carbon-based solid acids. The latter allow to overcome the problems of separation encountered with homogeneous catalysis. The mechanism first involves the isomerization of xylose into xylulose under Lewis acid-type catalysis, and the subsequent dehydration of xylulose into furfural under Brønsted acid-type catalysis (Scheme 47).

**Scheme 48:**
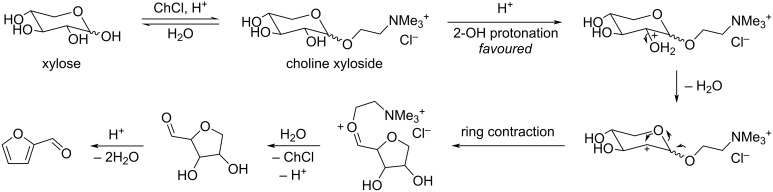
Mechanism for xylose dehydration to furfural through a choline xyloside intermediate.

Humins are naturally formed in all processes involving the acid-catalyzed degradation of carbohydrates [164–165]. Attempts to improve the balance between furfural and humins in high feed of starting xylose have been reported, for example by addition of choline chloride which is beneficial to the transformation of xylose to furfural in the presence of HCl (Scheme 48). The faster furfural formation rate is due to the formation of an intermediate choline xyloside which undergoes easier dehydration than xylose itself [166].

The examples given in the following sections illustrate the extremely vast range of reported transformations [160,167–176] of furfural toward useful biobased functional compounds or intermediates with applications in fuels, polymer chemistry and fine organic synthesis (CH activation, Piancatelli rearrangement, etc.).

**Scheme 49:**
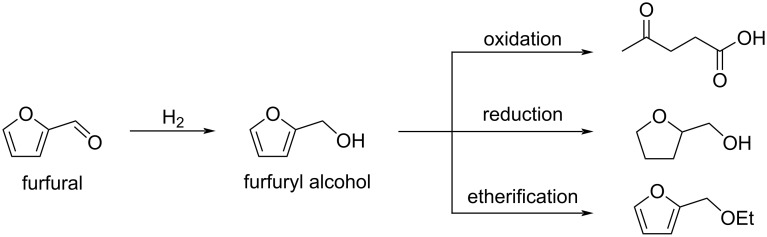
Conversion of furfural to furfuryl alcohol and its derivatives.

**Conversion of furfural to alcohols, aldehydes and ketones:** One of the most important transformations of furfural is its hydrogenation to produce furfuryl alcohol. This latter is a versatile intermediate for the production of resins, coatings, polymers and used as a solvent. It can also be converted into other chemicals through oxidative cleavage, over-reduction and etherification (Scheme 49) [177].

**Scheme 50:**
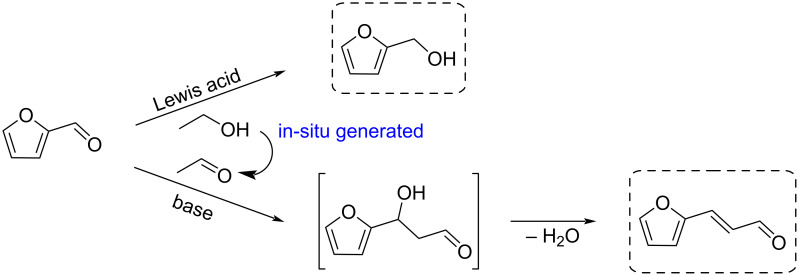
Conversion of furfural to furfuryl alcohol and 3-(2-furyl)acrolein.

Zhang et al. reported the use of Lewis acid–base bifunctional catalyst (Zr-β zeolite and K_2_CO_3_) in the Meerwein–Ponndorf–Verley reduction of a concentrated furfural solution (17.3–80.5 wt % in ethanol) combined with in situ cross-aldol condensation with acetaldehyde and crotonization. Ethanol was used as hydrogen donor for the Meerwein–Ponndorf–Verley reduction, while acetaldehyde is generated in situ by ethanol oxidation (Scheme 50). The equilibrium allows furfural to be simultaneously converted into furfuryl alcohol and 3-(2-furyl)acrolein in one pot through a redox reaction. The highest mass conversion rate of furfural can reach up to 95.3 g·L^−1^·h^−1^ [178].

**Scheme 51:**
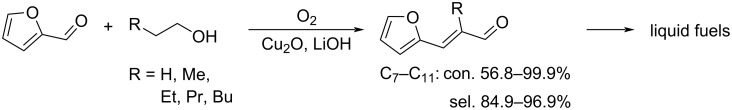
The aerobic oxidative condensation of biomass-derived furfural and linear alcohols.

While furfural oxidation is an important way to reach C_4_ platforms such as HFO [(see section 5-Hydroxy-2(5*H*)-furanone (HFO)], interesting C_5_ platforms can also be obtained. An example is the work by Shi et al. who reported the aerobic oxidative condensation of furfural with linear alcohols using Cu_2_O–LiOH catalytic system or under oxygen, thus achieving a carbon-chain increase from C_5_ to C_7–11_ with potential applications in the field of liquid fuels. The conversion of furfural is up to 99.9%, while the selectivity of the corresponding aldehyde is up to 96.9%. The catalyst could be recovered and reused five times without significant loss of activity (Scheme 51) [179].

**Scheme 52:**
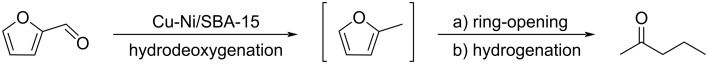
The single-step synthesis of 2-pentanone from furfural.

Furfural can be converted to 2-pentanone via simultaneous hydrodeoxygenation, ring-opening, and hydrogenation reactions under catalysis by a bimetallic Cu–Ni/SBA-15 catalyst in a fixed-bed continuous flow reactor. The moderate acidity and the hierarchical tube-type porous nature of SBA-15, as well as the adsorption energy on the surface of 10Cu–5Ni/SBA-15 catalyst, are the key parameters for giving 2-pentanone with high selectivity (78%) (Scheme 52) [180].

**Scheme 53:**
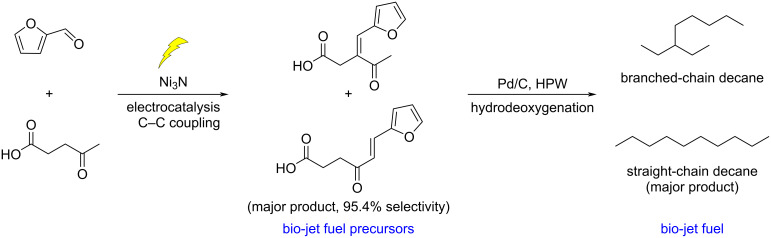
Electrocatalytic coupling reaction of furfural and levulinic acid.

Wang developed a new route for the production of jet fuel precursors through the electrocatalytic highly selective coupling reaction of furfural and levulinic acid. The Ni^2+^ species serves as the active site for the coupling process (Scheme 53) [181]. A similar strategy involving phenolic compounds and furfural allowed the production of bicyclic alkanes, used as fuel additives, by hydrodeoxygenation catalyzed by iridium/rhenium oxide supported on silica [182].

**Scheme 54:**
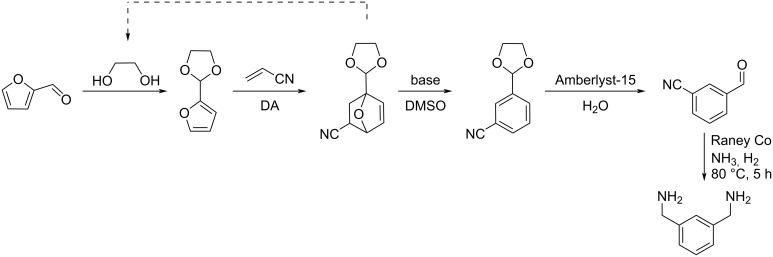
Conversion of furfural to *m*-xylylenediamine.

**Conversion of furfural to amines:** The synthesis of *m*-xylylenediamine from furfural and acrylonitrile through a Diels–Alder/aromatization sequence was developed by Wischet and Jérôme [183]. 2-(Furan-2-yl)-1,3-dioxolane with higher reactivity was generated through the acetalization of furfural with ethylene glycol, and converted to Diels–Alder cycloadducts in the presence of acrylonitrile at 60 °C. Treatment of the Diels–Alder adduct in basic conditions allowed the rearomatization of the system in favor of *meta* isomer. Acidic cleavage followed by reductive amination afforded *m*-xylylenediamine (Scheme 54).

**Scheme 55:**
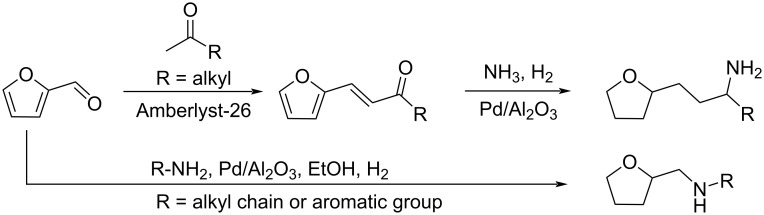
Conversion of furfural to tetrahydrofuran-derived amines.

Tetrahydrofuran-derived amines were prepared from furfural via a one-pot two-step reaction. The condensation of furfural with ketones over Amberlyst-26 as catalyst produced intermediate furan-derived enones. Subsequent reductive amination was performed in the presence of ammonia or amines and Pd/Al_2_O_3_ under H_2_ pressure, providing the tetrahydrofuran-derived amines [184]. The same catalytic system could allow the one-pot preparation of tetrahydrofuran-derived secondary and tertiary amines from furfural and amines with total reduction of the aromatic ring, as reported by Pera-Titus and De Oliveira Vigier (Scheme 55) [185].

**Scheme 56:**
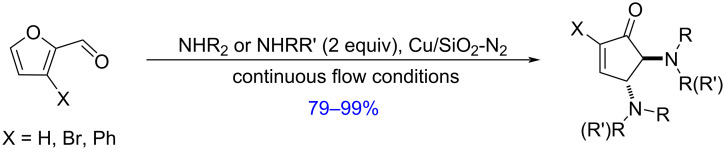
Formation of *trans*-4,5-diamino-cyclopent-2-enones from furfural.

A 2018 study reported the aza-Piancatelli reaction of furfural and secondary amines in deep eutectic solvent based on the ChCl–urea mixture furnishing *trans*-4,5-diamino-cyclopent-2-enones and 2,4-diaminocyclopent-2-enones [186]. In 2021, Coelho and Afonso prepared a silica-supported copper catalyst (Cu/SiO_2_-N_2_) by impregnation with copper nitrate, and used it for the reaction of furfural with secondary amines (alkyl, cyclic and aromatic) to produce *trans*-4,5-diaminocyclopent-2-enones in hexane/isopropanol solvent mixture under continuous flow conditions (Scheme 56). The scope could be extended to 2-substituted *trans*-4,5-diaminocyclopent-2-enones by using 3-substituted furfurals [187]. A copper triflate-based analogous strategy was also reported [188].

**Scheme 57:**
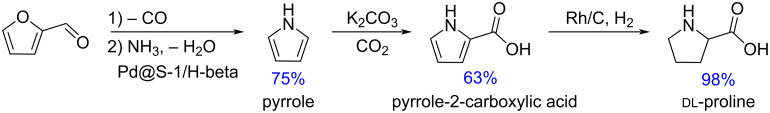
Production of pyrrole and proline from furfural.

**Conversion of furfural to pyrrole and proline:** Zhou and Yan reported a route bridging commercial biomass feedstock with high-value *N*-containing chemicals through pyrrole as a hub molecule. Furfural was converted into pyrrole in 75% yield via tandem decarbonylation–amination reactions over tailor-designed Pd@S-1 and H-beta zeolite catalytic system, exhibiting high resistance to coking in presence of NH_3_. Subsequent carboxylation with CO_2_ and hydrogenation over Rh/C as catalyst led to racemic proline, an important precursor for pharmaceuticals, in quantitative yield. ᴅ-Proline was then obtained in 50% yield and 99% ee after kinetic resolution using *Escherichia coli* (Scheme 57) [189].

**Scheme 58:**
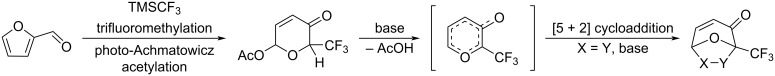
Synthesis of 1‑(trifluoromethyl)-8-oxabicyclo[3.2.1]oct-3-en-2-ones from furfural.

**Other reactions:** Yamamoto synthesized 6-hydroxy-2-(trifluoromethyl)-2*H*-pyran-3(6*H*)-ones from furfural via trifluoromethylation using the Ruppert–Prakash reagent (TMSCF_3_), followed by a photo-Achmatowicz reaction. Then, through acetylation and subsequent base-mediated oxidopyrylium [5 + 2] cycloaddition reactions, 1-(trifluoromethyl)-8-oxabicyclo[3.2.1]oct-3-en-2-ones were prepared (Scheme 58) [190].

**Scheme 59:**
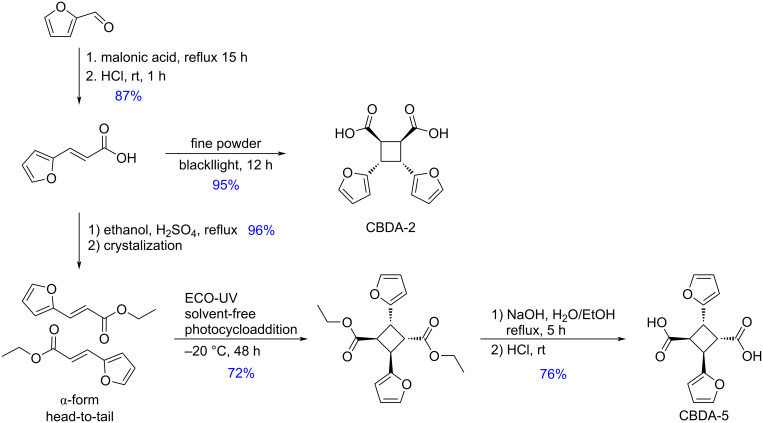
Conversion of furfural to furfural-derived diacids.

Chu and Webster reported the photocatalyzed synthesis of furfural-derived diacids from furfural [191–192]. Furfural first was reacted with malonic acid to produce *trans*-3-(2-furyl)acrylic acid in a Knoevenagel reaction, which was then converted into cyclobutane-containing diacids through a [2 + 2] photoreaction. *cis*-3,4-Di(furan-2-yl)cyclobutane-1,2-dicarboxylic acid (CBDA-2) prepared under blacklight UV irradiation was proven to be thermally, chemically and sunlight stable. The esterification of *trans*-3-(2-furyl)acrylic acid with ethanol, provided (1α,2α,3β,4β)-2,4-di(furan-2-yl)cyclobutane-1,3-dicarboxylic acid (CBDA-5) via a solvent-free [2 + 2] photodimerization from ethyl 2-furanacrylate and subsequent hydrolysis (Scheme 59).

**Scheme 60:**
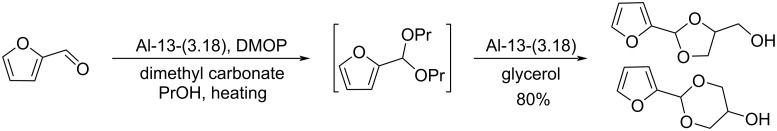
A telescope protocol derived from furfural and glycerol.

Furfural was employed as starting scaffold in a protocol developed to form acetals as fuel additives. The acyclic acetal is generated in situ from crude furfural with propanol in dimethyl carbonate using a nanoporous aluminosilicate material Al-13-(3.18) as catalyst, and then undergoes transacetalization with glycerol to produce a mixture of corresponding dioxane and dioxolane products (Scheme 60) [193].

**Scheme 61:**
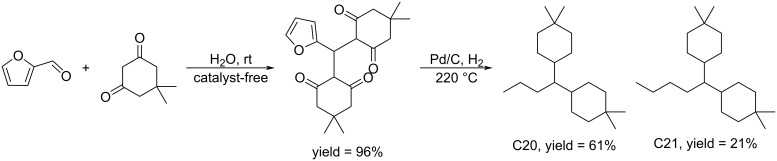
A tandem cyclization of furfural and 5,5-dimethyl-1,3-cyclohexanedione.

Another strategy was proposed to synthesize potential aviation biofuels from furfural and 5,5-dimethyl 1,3-cyclohexanedione in water or under solvent-free conditions (Scheme 61). The obtained hydrocarbons exhibited higher density (0.78–0.88 g/cm^3^) and higher net heat of combustion (44.0–46.0 MJ/kg) than some commonly commercial fuels [194].

**Scheme 62:**
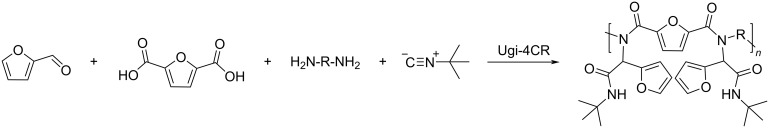
A Ugi four-component reaction to construct furfural-based polyamides.

Multicomponent reactions involving the reactivity of furfural are also noteworthy. A Ugi four-component polymerization was developed to synthesize photoluminescence-active polyamides from renewable furfural and 2,5-furandicarboxylic acid with diamine and isocyanide (Scheme 62). The intramolecular hydrogen bonding between the secondary amide and furan oxygen atom promote the strong fluorescence of the polyamides. These furfural-based polyamides possess unusual intrinsic luminescence and high and selective affinity towards Fe^2+^ and Fe^3+^ metal ions, which could be used as fluorescent probes in the biomedical field [195].

**Scheme 63:**
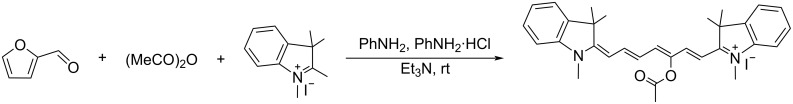
One-pot synthesis of γ-acyloxy-Cy7 from furfural.

Heptamethine cyanine Cy7 is a very important class of intermediates in the fields of biology and photomedicine. The introduction of substituents to the seven-methine carbon chain of Cy7 influences its optical properties and ability to interact with biomolecules. A one-pot synthesis of γ-acyloxy-Cy7 from renewable furfural, simple anhydrides, and a quaternary ammonium salt under smooth conditions was reported (Scheme 63). Partial γ-acyloxy-Cy7 possesses a high fluorescence quantum yield and high photothermal conversion efficiency in PBS, two pivotal parameters in fluorescence imaging and image-guided photothermal therapy [196].

**Scheme 64:**
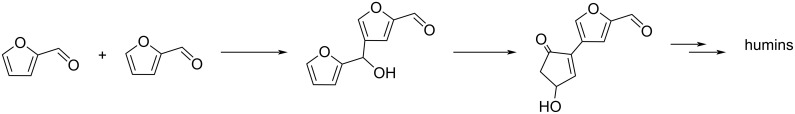
Dimerization–Piancatelli sequence toward humins precursors from furfural.

A dimerization of furfural has been found to occur at the early stage of formation of humins, arising from a nucleophilic attack of a furan carbon atom of one furfural molecule onto the aldehyde of a second one, giving 2-(4-furfur-2-al)-4-hydroxy-2-cyloepenten-1-one (Scheme 64), resulting from a Piancatelli rearrangement. This latter can further evolve towards more complex humin precursors by etherification and aldol reactions [197].

#### Cyclopentanone (CPN)

**Scheme 65:**
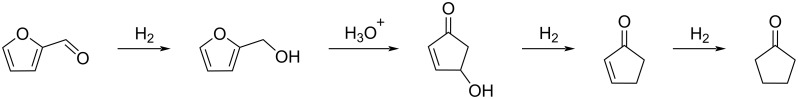
Conversion of furfural to CPN.

Cyclopentanone (CPN) can be prepared by catalytic hydrogenation and Piancatelli rearrangement of furfural (Scheme 65). This is a biobased alternative to the fossil-based process relying on the intramolecular decarboxylative cyclization of adipic acid. In the biobased reaction from furfural to CPN, the key points influencing the selectivity and yield are the choice of metal catalyst, hydrogen pressure and supporting acidic materials [168,198].^.^When carried out in aqueous medium, the reaction was found to provide better selectivity by limiting the generation of other hydrogenation products than in organic solvents [199]. Since this new route to CPN from furfural was reported in 2012, many catalysts have been proposed for promoting this reaction. Recent works include heterogeneous systems based on cobalt [200], nickel [201], copper [202] or palladium [203]. Biobased CPN can also be directly obtained from C-5 carbohydrates. For example, Zhang and Li recently reported a two-step route directly from xylose (or hemicellulose) going through cyclopentenone as intermediate. A subsequent hydrodeoxygenation/dehydrogenation sequence led to cyclopentadiene, or methylcyclopentadiene upon additional aldol condensation with in situ generated formaldehyde, both highly useful intermediates for the production of high-energy-density rocket fuels and polymers [204].

**Conversion of CPN to hydrocarbons:** CPN can be used as a precursor to produce high-octane diesel or jet fuel additives through aldol condensation/hydrogenation sequences. The acyclic or cyclic chain hydrocarbons can be formed under hydrodeoxygenation conditions after aldol extension of the C–C chain. Dimers and trimers can be obtained from CPN by self-condensation and converted to C_10_ and C_15_ alkane fuels (bicyclopentane, tricyclic alkane and spirocyclic alkane) through hydrodeoxygenation [205–210].

**Scheme 66:**
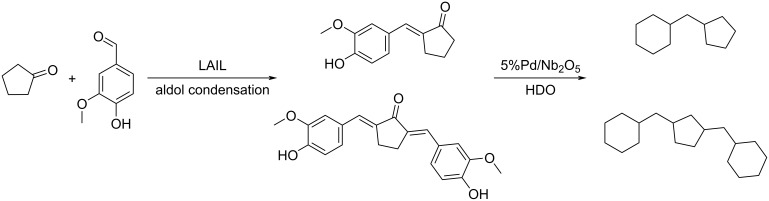
Synthesis of jet fuels range cycloalkanes from CPN and lignin-derived vanillin.

A cycloalkane with a density of 0.89 g/cm^3^ and a freezing point below −60 °C was prepared from CPN and lignin-derived vanillin. An overall yield of 95.2% of mono- and di-condensation products was obtained at 100 °C over ethanolamine lactate ionic liquid (LAIL). The condensation products were converted into jet fuels range cycloalkanes through aqueous phase hydrodeoxygenation (HDO) by using 5% Pd/Nb_2_O_5_ catalyst (Scheme 66) [211].

**Scheme 67:**
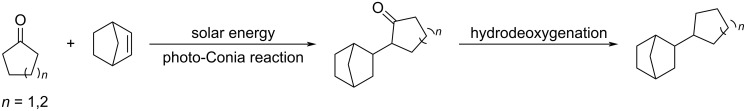
Solar-energy-driven synthesis of high-density biofuels from CPN.

A new route to novel high-performance aviation fuels driven by solar energy such as non-symmetrical polycyclic hydrocarbons has been proposed by using a photosensitized Conia reaction of ketones and norbornene (Scheme 67) [212]. The hydrocarbons derived from CPN and cyclohexanone endow high density of 0.935 and 0.941 g mL^−1^, respectively, much higher than conventional aviation kerosene (0.78 g mL^−1^).

**Scheme 68:**
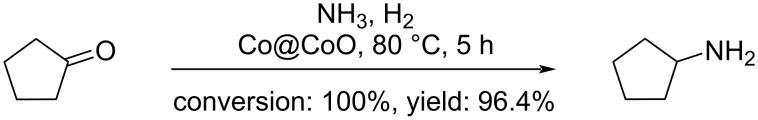
Reductive amination of CPN to cyclopentylamine.

**Conversion of CPN to cyclopentylamines and alcohols:** Gong and Wang revealed the potential of cobalt oxide catalysts for the production of primary amines via reductive amination of CPN (Scheme 68) [213]. CoO species with oxygen vacancies on the surface have a unique ability to dissociate NH_3_ and generate hydrogen-like species (NH_2_^δ−^), thus accelerating the ammonolysis of the Schiff base to produce the corresponding primary amine. Cyclopentylamine was generated from CPN in 96% yield over a core‐shell‐structured Co@CoO catalyst at 80 °C under 0.3 MPa NH_3_ and 2 MPa H_2_. The reaction could be carried for 10 catalytic cycles without deactivation.

**Scheme 69:**
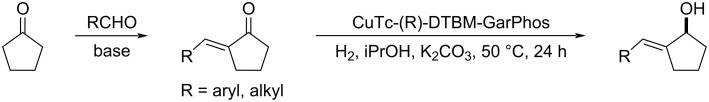
Asymmetric hydrogenation of C=O bonds of exocyclic α,β-unsaturated cyclopentanones.

Zhang developed a transition-metal copper-catalyzed chemoselective asymmetric hydrogenation of the carbonyl group in exocyclic α,β-unsaturated cyclopentanones. Chiral exocyclic allylic pentanols (a versatile scaffold for bioactive molecules) with up to 99% yield and 96% ee were prepared (Scheme 69). The large electron-donating groups of 3,5-di-*t*ert-butyl-4-methoxyphenyl (DTBM) improved the activities of the Cu^I^–thiophene-2-carboxylate catalyst (CuTc) while the use of (*R*)-DTBM-GarPhos as the chiral ligand led to excellent enantioselective induction during the hydrogenation step. Higher H_2_ pressure could promote the regeneration of Cu–H species [214].

#### Levulinic acid (LEV)

**Scheme 70:**
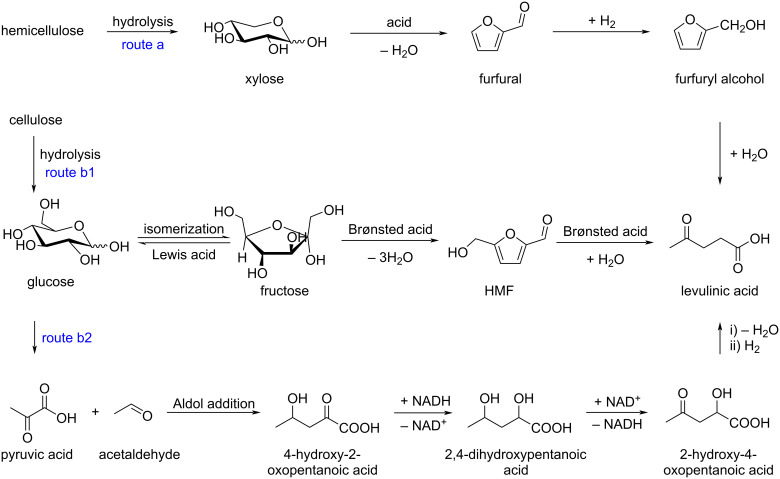
Preparation of levulinic acid via the C_5_ route (route a) or C_6_ route (routes b1 and b2).

Levulinic acid is a biodegradable C_5_ carboxylic acid with high potential as a platform chemical in the field of polymers, fuels, pharmaceuticals or high added value chemicals [215–223]. Its first commercial production began in the 1940s. Its preparation from biomass involves several different possible pathways. One is the C_5_ sugar route starting from xylose as an example, relying on acid hydrolysis of hemicellulose, dehydration to furfural, hydrogenation into furfuryl alcohol and final hydrolysis to levulinic acid (Scheme 70, route a). The second route is the acid-catalyzed multistep conversion of polysaccharides into C_6_ monosaccharides (glucose and fructose), followed by dehydration to 5-HMF, and then rehydration into levulinic acid (Scheme 70, route b1). It is now preferred considering raw material costs, process efficiency and market demand. Alternatively, C_6_ monosaccharides can also be fermented to produce pyruvic acid and acetaldehyde, which combine by an aldol reaction and lead to levulinic acid by isomerization and hydrogenation (Scheme 70, route b2).

**Scheme 71:**
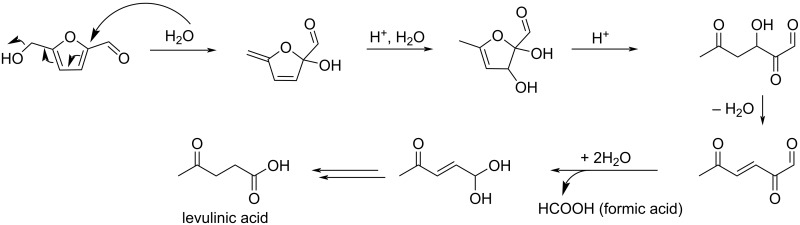
Mechanism of the rehydration of HMF to levulinic acid and formic acid.

The accepted mechanism of the rehydration of HMF under acidic conditions leading to the formation of levulinic acid and formic acid proposed by Horvat [224] is depicted in Scheme 71.

**Scheme 72:**
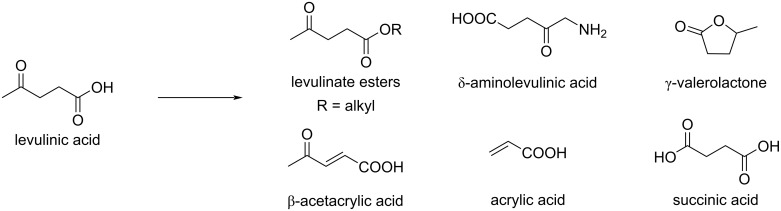
Important levulinic acid-derived chemicals.

Levulinic acid can be converted into various derivatives, such as esters, amides, lactones, acrylic acid, etc. (Scheme 72) and is therefore a key intermediate in the synthesis of pharmaceuticals, agrochemicals, and other specialty chemicals. Levulinate esters, used as a solvent or as biofuels, are obtained by esterification under acid catalysis, e.g., with a sulfuric acid-modified ultra-stable Zeolite Y (SY) [225] or Pd_NPs_-TiO_2_/Al_2_O_3_/SiO_2_ [226].

**Scheme 73:**
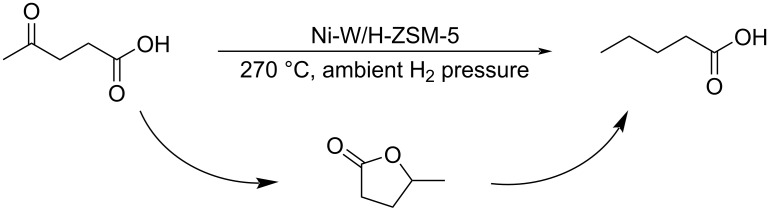
Direct conversion of levulinic acid to pentanoic acid.

**Conversion to other carboxylic acids:** Valeric acid (VA) and valerate esters (VEs), also named pentanoic acid or esters, are targeted as fuel additives and value-added chemicals. They are obtained by hydrogenation of levulinic acid using a wide range of noble or non-noble metal catalysts [227]. For example, treatment of levulinic acid under vapor phase in a continuous fixed-bed with bifunctional mesoporous SBA-15 doped with metals such as Nb, Ti, and Zr having hydrogenation centers and acidic sites led to VA, with GVL and alkyl levulinates obtained as by-products [228]. Xue and Wu reported the electrocatalytic reduction of levulinic acid to VA using a nanocrystalline PbO–In_2_O_3_ composite material as working electrode in an H-type electrolytic cell [229], while Shen used bimetallic In–Pb electrocatalysts [230], and Tao applied a β-PbO/Pb electrode [231]. Another example reported by the Akula group developed a direct conversion of levulinic acid to VA at 270 °C and ambient H_2_ pressure in a continuous flow fixed-bed reactor (Scheme 73) in the presence of Brønsted-acidic material H-ZSM-5-supported W-modified Ni catalyst. The mechanism involves a ring opening of GVL at the Brønsted acidic sites, while the β-proton abstraction occurs on the support surface basic sites [232].

**Scheme 74:**
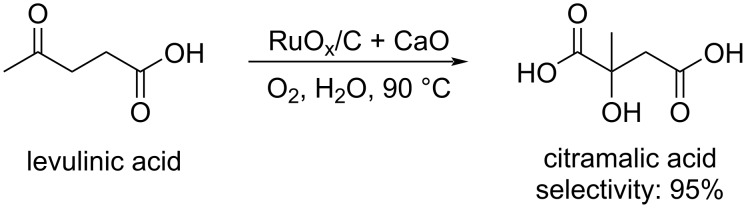
Catalytic aerobic oxidation of levulinic acid to citramalic acid.

Catalytic aerobic oxidation of levulinic acid towards dicarboxylic acids is a way to obtain monomers for the manufacture of biobased polymers. For example, citramalic acid was obtained with up to 95% selectivity over acetic acid and succinic acid using a low-cost reusable heterogeneous RuO_x_/C and CaO binary catalyst at 90 °C (Scheme 74) [233]. Levulinic acid can also be efficiently converted into succinic acid [234–235].

**Scheme 75:**
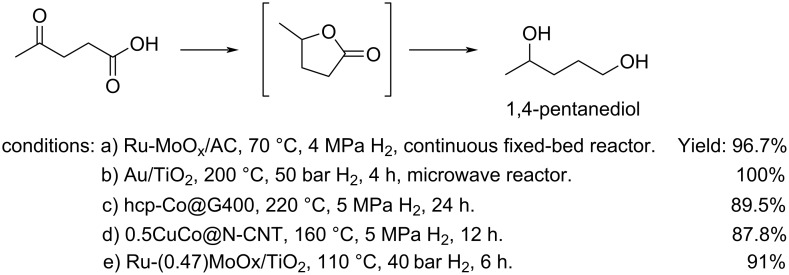
Conversion of levulinic acid to 1,4-pentanediol (a) see ref. [236]; b) see ref. [237]; c) see ref. [238]; d) see ref. [239]; e) see ref. [240]).

**Conversion to 1,4-pentanediol or 2-butanol:** 1,4-Pentanediol, a useful raw material for the synthesis of polyesters, biofuels and other chemicals, can be obtained from LEV by catalytic hydrogenation. Non-noble metal hydrogenation catalysts are generally more challenging in promoting the conversion of levulinic acid to 1,4-pentanediol because they are more prone to corrosion compared with noble-metal catalysts. This has encouraged the design of highly active and more stable heterogeneous non-noble metal-based catalysts able to tolerate the presence of carboxylic acids, such as those listed in Scheme 75 [236–240].

**Scheme 76:**
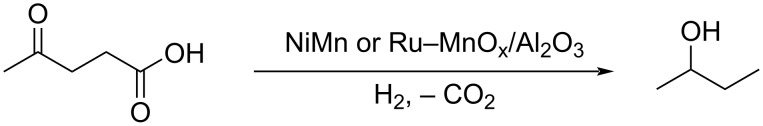
Selective production of 2-butanol through hydrogenolysis of levulinic acid.

In 2021, Wang reported the high efficiency of the non-precious bimetallic catalyst Ni–Mn in the selective hydrogenolysis of levulinic acid to 2-butanol in 82.5% yield under 5 MPa H_2_ at 240 °C (Scheme 76) [241]. The high basicity of the Ni–Mn catalyst results in a good affinity with the C=O or COOH groups of levulinic acid-derived molecules. The same group later reported a more active heterogeneous catalyst, Ru–MnO*_x_*/Al_2_O_3_, providing 2-butanol with yields up to 98.8% [242].

**Conversion to 2-methyltetrahydrofuran:** 2-Methyltetrahydrofuran is a promising biofuel with high energy density, high stability and low miscibility with water. Investigations of the catalytic transformation of levulinic acid to 2-methyltetrahydrofuran over bimetallic NiCo/γ-Al_2_O_3_ catalyst or Ni phyllosilicates have shown that high temperature, high pressure and low selectivity are still important limitations [243–244].

**Scheme 77:**
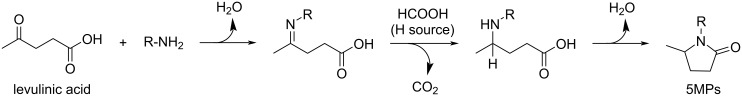
General reaction pathways proposed for the formation of 5MPs from levulinic acid.

**Reductive amination of levulinic acid:** The reductive amination of levulinic acid and its esters leads to the formation of *N*-substituted 5-methyl-2-pyrrolidinones (5MPs) which are used in many fields including pharmaceutical and dye industries. Zhang reviewed the synthesis of 5MPs from levulinates and different substituted amines under metal catalysis or catalyst-free conditions, using hydrosilane, formic acid, hydrogen, MeOH or HCOOH/Et_3_N as reducing sources [245]. The type and recyclability of catalysts, the nature of supports, reaction conditions, and reactors play important roles in the sustainability of the conversion of levulinic acid to 5MPs. A well-accepted route from levulinic acid to 5MPs is shown in Scheme 77.

Recent examples of LEV to 5MPs conversions include the use of a ruthenium ion supported on ionic liquid immobilized into graphene oxide (Ru@GOIL), polyvinylpyrrolidone-stabilized metal nanoparticles (Ir–PVP), palladium catalysts synthesized via wet impregnation or Co–M bimetallic C–N doped catalyst (Co–Zr@Chitosan-20) [246–249]. An efficient method for preparing *N*-free 5-methyl-2-pyrrolidone from levulinic acid was developed by Li and Yang using formamide as nitrogen source and formic acid as hydrogen source via transfer hydroamination/amidation and cyclization under quasi solvent-free and catalyst-free conditions [250]. Song and Han designed TiO_2_ nanosheets-supported Pt nanoparticles (Pt/P-TiO_2_) as porous heterogeneous catalysts with high acidity and low electron density at the Pt sites. Using H_2_ as the hydrogen source, such catalysts exhibited high activity for the conversion of LEV to 5MP, being able to promote the formation of a variety of *N*-substituted pyrrolidones at room temperature in excellent yields [251]. In 2022, Liu and Wei synthesized *N*-substituted 5-methyl-2-pyrrolidinones from levulinic acid/esters and various amines in high yields (80–99%) over 0.05 mol % of an N-doped mesoporous carbon-supported Ru catalyst (Ru/NMC) at 120 °C and 1.5 MPa H_2_ [252].

**Scheme 78:**
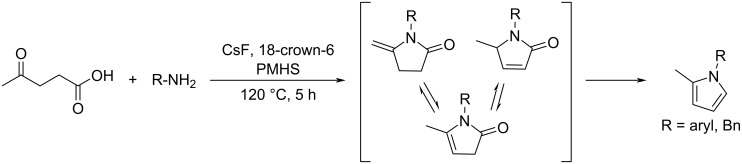
Selective reductive amination of levulinic acid to *N*-substituted pyrroles.

Liu and Wang reported the selective reductive amination/cyclization of levulinic acid with primary arylamines in the presence of polymethylhydrosiloxane (PMHS) to *N*-substituted arylpyrroles in a yield range of 35–93%. The 18-crown-6 ether coordinates the cesium ion, thus increasing the nucleophilicity of the fluoride anion. The F^−^ anion contributes to the activation of the hydrosilane during the reduction step (Scheme 78) [253].

**Scheme 79:**
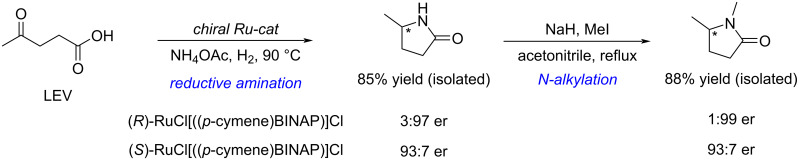
Reductive amination of levulinic acid to chiral pyrrolidinone.

The asymmetric reductive amination of levulinic acid with ammonium acetate can be achieved using commercially available chiral Ru/bisphosphine catalysts, giving 5MPs with excellent enantioselectivity (97% ee) and isolated yield (85%). The chiral 5MPs can then be further functionalized by *N*-alkylation without loss of enantioselectivity (Scheme 79) [254].

**Scheme 80:**
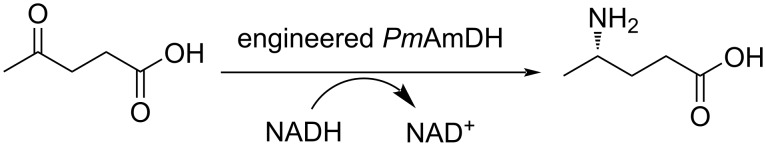
Reductive amination of levulinic acid to non-natural chiral γ-amino acid.

Xu and Zheng prepared a non-natural chiral γ-amino acid from levulinic acid in biocatalytic conditions using an engineered amine dehydrogenase *Pm*AmDH_I80T/P224S/E296G,_ obtained from a thermophilic bacterium (*Pm*AmDH) by direct evolution in the presence of NaDH. This process leads to (*S*)-4-aminopentanoic acid with >99% ee and 90% yield at 40 °C, and an 18-fold increase in the catalytic efficiency compared with the wild-type enzyme (Scheme 80) [255].

**Scheme 81:**
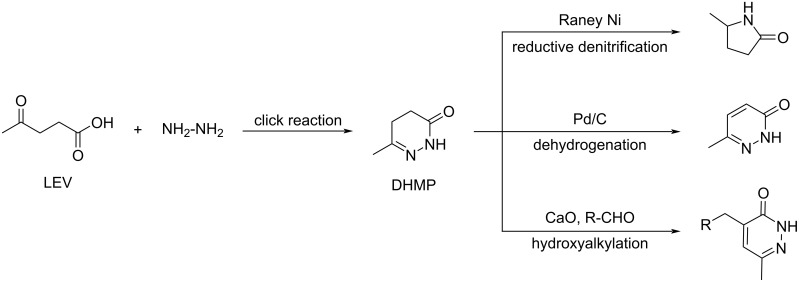
Nitrogen-containing chemicals derived from levulinic acid.

Huang and Lu reported that levulinic acid and hydrazine hydrate can react without a catalyst and in quantitative yield within few minutes in water at room temperature to form the 4,5-dihydro-6-methylpyridazin-3(2*H*)-one (DHMP) platform, in a fashion similar to a click reaction. DHMP can be further selectively converted into pyrrolidine and (alkyl)-pyridazinone through reductive denitrification, dehydrogenation and hydroxyalkylation reactions (Scheme 81) [256].

#### γ-Valerolactone (GVL)

**Scheme 82:**
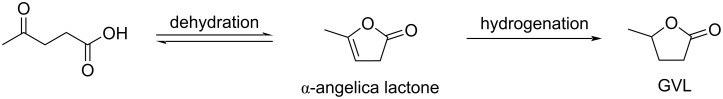
Preparation of GVL from levulinic acid by dehydration and hydrogenation.

γ-Valerolactone (GVL) is a representative cyclic ester derived from levulinic acid that is commonly used as a biobased solvent with high boiling point (207 °C) able to replace NMP or DMF, and as a chemical platform in various industrial applications [257]. Catalytic production of GVL relies on a dehydration–hydrogenation sequence from levulinic acid via an α-angelica lactone intermediate (Scheme 82) [258]. This has been a hot topic in the past decade with many efficient strategies using various catalysts involving metals such as platinum [259–260], ruthenium [261], titanium [262] or copper [263] as well as other bimetallic species (Ni/Ru, Ni/Mo, Ni/Zn, etc.) [264–268] or in alternative solvents [269].

**Scheme 83:**
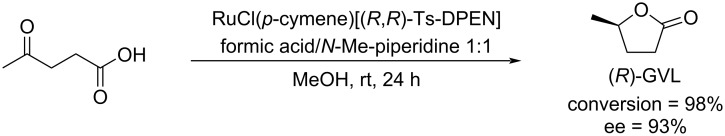
Ruthenium-catalyzed levulinic acid to chiral γ-valerolactone.

**Scheme 84:**
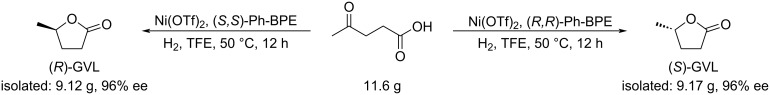
Catalytic asymmetric hydrogenation of levulinic acid to chiral GVL.

Studies targeting enantiomerically enriched γ-valerolactone have been also reported. For example, the Bhanage group reported the use of a ruthenium-catalyzed asymmetric transfer hydrogenation of levulinic acid to (*R*)-γ-valerolactone with 93% enantiomeric excess (ee). Using *N*-methylpiperidine with formic acid as an available hydrogen donor and Noyori’s chiral catalyst (Ru-(*R*,*R*)-TsDPEN), this route led to 98% LEV conversion. The direct transformation of LEV obtained from a “raw biomass” resource such as rice husk and corn straw, to optically active GVL was performed by asymmetric hydrogenation with good enantioselectivity (82% ee) (Scheme 83) [270]. Deng and Fu reported the nickel-catalyzed asymmetric hydrogenation of aliphatic ketoacids to chiral γ- and δ-alkyl-substituted lactones. A robust and highly active homogeneous chiral nickel–phosphine catalyst (Ni(OTf)_2_ with (*S*,*S*)-Ph-BPE) as chiral ligand afforded the chiral lactones in up to 98% yield with 95% ee. A ten-gram scale reaction from LEV to chiral GVL (96% ee) was achieved in the presence of only 0.02 mol % catalyst loading (Scheme 84). (*S*)-GVL could be also obtained by using (*R,R*)-Ph-BPE [271]. The same group used a nickel–phosphine complex [272] or a combination of nickel phosphine complex and metal triflate, both in solvent-free conditions [273].

**Scheme 85:**
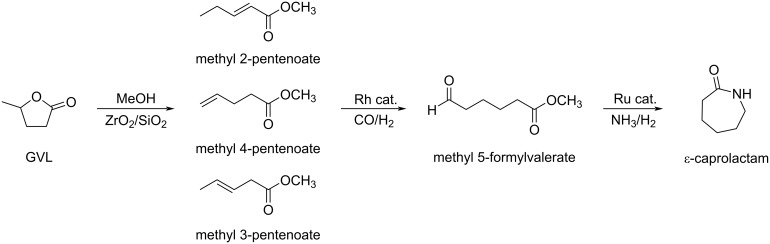
Three steps synthesis of *ε*-caprolactam from GVL.

**Uses of GVL in polymer chemistry:** GVL is a useful building block for the synthesis of polyesters and other polymers. Kalevaru and de Vries reported the ring-opening reaction of GVL with methanol over ZrO_2_/SiO_2_ as catalyst in a continuous gas-phase process with 95% conversion towards methyl 2-, 3-, and 4-pentenoate (81% in favor of M4P). Methyl 4-pentenoate was then converted into methyl 5-formylvalerate with 90% selectivity, leading to subsequent generation of *ε*-caprolactam (the monomer of nylon-6) via reductive amination and ring closure (Scheme 85) [274].

**Scheme 86:**
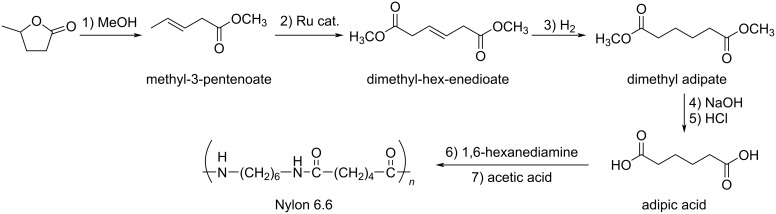
Multistep synthesis of nylon 6,6 from GVL.

Kim and Han developed a protocol for the synthesis of nylon 6.6 from GVL. After esterification of GVL to methyl-3-pentenoate, subsequent metathesis of methyl-3-pentenoate afforded dimethyl hexenedioate. Upon hydrogenation, this alkene was converted into dimethyl adipate, which, under hydrolysis provided adipic acid. The final step was the polymerization of adipic acid to nylon 6,6 in the presence of 1,6-hexanediamine (Scheme 86) [275].

**Scheme 87:**
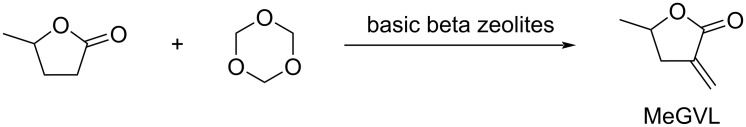
Preparation of MeGVL by α-alkylation of GVL.

GVL can be transformed into α-methylene-γ-valerolactone (MeGVL), an interesting monomer, that can provide a material with properties similar to the widely used poly(methyl)methacrylate when subjected to visible-light-induced polymerization. Under the action of a base like a beta (Si/Al = 150) zeolite, MeGVL can be formed by nucleophilic attack of the carbon atom at α-position of GVL on formic acid, arising from the decomposition of trioxane (Scheme 87) [276].

**Scheme 88:**
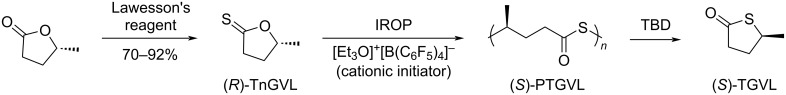
Ring-opening polymerization of five-membered lactones.

Hong reported the conversion of GVL to polythioesters via the isomerization-driven ring-opening polymerization (IROP) of thionolactone intermediates (TnGVL) using [Et_3_O]^+^[B(C_6_F_5_)_4_]^−^ as cationic initiator [277–278]. The enantiopure TnGVL undergoes an inversion of configuration during the IROP process through a S_N_2 ring-opening mechanism. The method provided a highly isotactic semi-crystalline thermoplastic (Scheme 88).

#### Ionic liquids from GVL

**Scheme 89:**
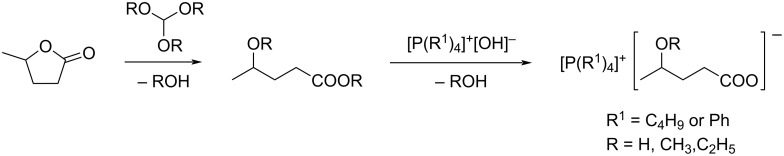
Synthesis of GVL-based ionic liquids.

Ionic liquids are innovative environmentally benign reaction media due to their extremely low vapor pressure and easily tunable properties. In 2018, Mika synthesized GVL-based ionic liquids from GVL and tetrabutylphosphonium [TBP] or tetraphenylphosphonium [TPP] hydroxides in excellent yield (Scheme 89). These phosphonium-based ionic liquids showed better thermal stability than their ammonium analogues. Their usefulness was illustrated in industrially important transformations such as the copper-catalyzed Ullmann-type coupling reaction [279] or the Sonogashira coupling of aryl iodides and functionalized acetylenes [280].

**Scheme 90:**
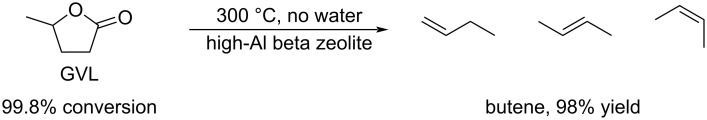
Preparation of butene isomers from GVL under Lewis acid conditions.

**GVL towards biofuels:** An important application of GVL is to produce butene by decarboxylation reaction for liquid fuels application. For example, Xin and Zhang reported a one-pot conversion of GVL to butene in 98% yield with 99.5% GVL conversion involving the catalytic decarboxylation of GVL at 300 °C in the presence of high aluminum content beta zeolite (Scheme 90) [281].

**Scheme 91:**
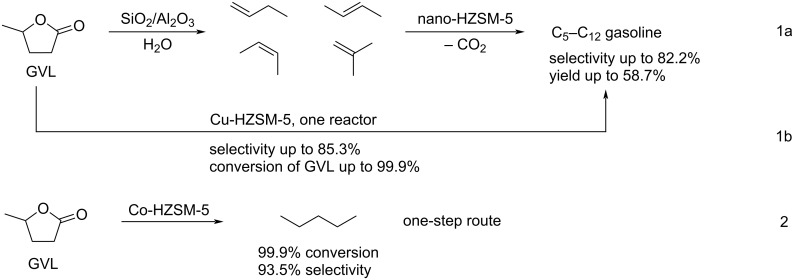
Construction of C_5_–C_12_ fuels from GVL over nano-HZSM-5 catalysts.

Zeng and Ding targeted C_5_–C_12_ gasoline range fuels from GVL in an integrated two-stage fixed-bed catalytic system. In this process, GVL was first converted to a mixture of butene isomers via a ring-opening–decarboxylation sequence, which then oligomerized to gasoline fuels over a nano-HZSM-5 zeolite catalyst (Scheme 91, 1a) [282]. The same team proposed a direct one-step Cu/HZSM-5 (Si/Al ratio of 15)-catalyzed selective conversion of GVL to C_5_–C_12_ hydrocarbon fuels (Scheme 91, 1b) or to pentane fuels (Scheme 91, 2) [283–284].

**Scheme 92:**
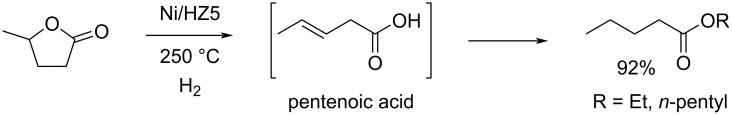
Preparation of alkyl valerate from GVL via ring opening/reduction/esterification sequence.

**Other reactions of GVL:** The Csp^3^–O bond of GVL can be cleaved leading to a pentenoic acid intermediate, which can then be hydrogenated into ethyl valerate. In this sequence, which is promoted by a H-ZSM-5 supported Ni catalyst, pentenoic acid (not isolated) was generated in situ and was rapidly hydrogenated and esterified to ethyl valerate with 92% selectivity in the vapor phase (Scheme 92) [285]. Bertero reported the one-pot production of pentyl valerate with high conversion (83.5%) and selectivity (87.8%) in liquid phase over a SiO_2_/Al_2_O_3_-supported Ni-based catalyst (Ni/SA-I) at 523 K under 10 bar of H_2_ [286]. Using SiO_2_/Al_2_O_3_-supported Pt-based catalysts with moderate metal loading (1%), a 90% yield of pentyl valerate was obtained with 100% conversion of GVL at 523 K and 10 bar of H_2_. This process resulted in a high productivity in pentyl valerate (300 mmol/g_M_·h) [287].

**Scheme 93:**
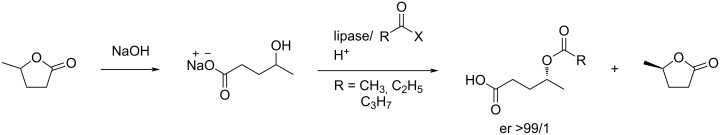
Construction of 4-acyloxypentanoic acids from GVL.

Alkaline hydrolysis of GVL produces 4-hydroxycarboxylic acid salts. These latter can be used in lipase-catalyzed stereoselective acylation of the 4-hydroxy group [288]. Using CALB (Novozym 435) as lipase, (*R*)-4-acyloxypentanoic acids were obtained in a moderate yield (<50%) from racemic GVL after acid hydrolysis of the intermediate salt, with (*R*)-GVL as by-product (Scheme 93).

**Scheme 94:**
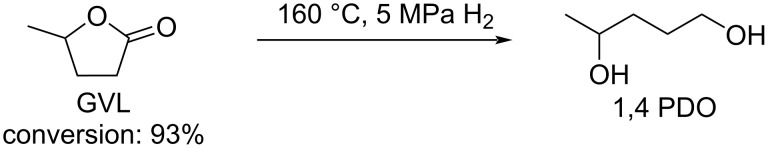
Synthesis of 1,4-pentanediol (PDO) from GVL.

The hydrogenation of GVL to pentanediol by bifunctional Cu_x_/Mg_3−x_AlO nanocatalysts (x = 0.5, 1, 1.5, 2) with excellent catalytic performance has been reported (Scheme 94). Thanks to synergistic effects of the well-dispersed active Cu nanoparticles and the relevant surface basic sites, the process provided 1,4-pentanediol (PDO) with a selectivity over 99% and a GVL conversion of 93% at 160 °C and 5MPa H_2_ [289]. Another highly selective and efficient protocol using a Cu/SiO_2_ triethoxyoctylsilane-modified catalyst was reported by Cappelletti [290].

**Scheme 95:**
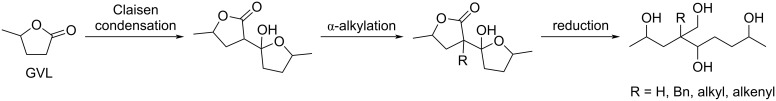
Construction of novel cyclic hemiketal platforms via self-Claisen condensation of GVL.

The self-Claisen condensation of GVL in the presence of *t*-BuOK at 25 °C provided a novel cyclic hemiketal platform in 85% yield. Alkylation and reduction of such cyclic hemiketals led to polyols with long chains ranging from C_10_ to C_21_ (Scheme 95) [291].

**Scheme 96:**
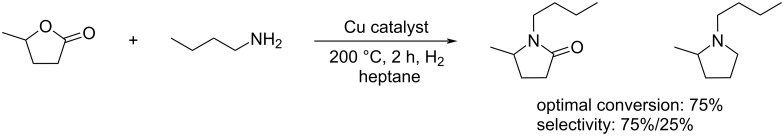
Copper-catalyzed lactamization of GVL.

*N*-Alkylpyrrolidones, which are promising solvent alternatives to NMP, can be derived from GVL. In an effort aiming at avoiding noble-metal catalysts and high hydrogen pressure, the Zaccheria group has applied a CuO/Al_2_O_3_ catalyst which allowed the conversion of GVL to *N*-alkylpyrrolidone solvents at a 10-bar hydrogen pressure (Scheme 96) [292].

### C_6_ biobased carbonyl platforms

#### 5-Hydroxymethylfurfural (HMF)

5-Hydroxymethylfurfural (HMF) arises from C_6_ carbohydrates (or their polymers) or crude lignocellulosic raw materials [293–298]. HMF has been added to the updated “Top potential value-added chemicals” list in 2010 by Bozell and Petersen [215] considering its functional versatility and wide range of applications in fine chemistry. Extensive recent literature publications and reviews demonstrate the importance of HMF in modern chemistry [299–302]. Its moderate thermal and chemical stability either under acidic or basic conditions is responsible for the formation of levulinic acid (see section Levulinic acid (LEV), Cannizzaro products (such as 2,5-dihydroxymethylfurfural (DHMF) and 5-hydroxymethylfuranoic acid (HMFA)), oligomerization or humins [303–304].

**Figure 4:**
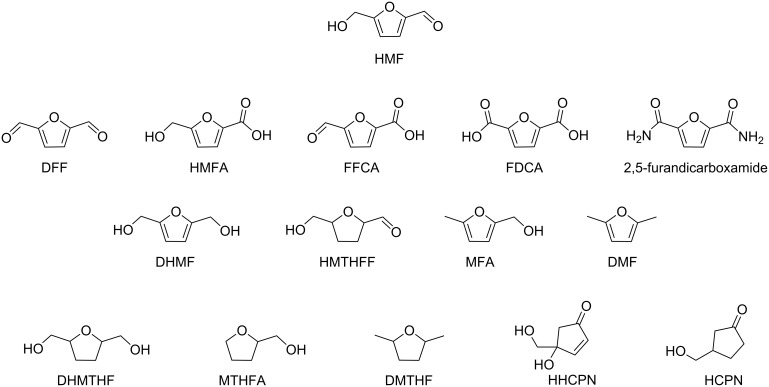
Main scaffolds obtained from HMF.

Regarding HMF oxidation, a major topic is the path towards new biobased polymers via the furandicarboxylic (FDCA) platform, when combined with diols or diamines (Figure 4) [8,305–311]. Other oxidation products such as diformylfuran (DFF), hydroxymethyl-2-furanoic acid (HMFA), 5-formyl-2-furancarboxylic acid (FFCA) are also interesting intermediates, as well as the corresponding diamide, resulting in numerous studies and reviews on HMF (Figure 4) [312–320]. Several HMF-derived monomers attest its versatile role in polymer chemistry [321–323]. On the reduction side, many useful chemical intermediates, such as dihydroxymethylfuran (DHMF), (hydroxymethyl)tetrahydrofurfural (HMTHFF), methylfurfurylalcohol (MFA), dimethylfuran (DMF), dihydroxymethyltetrahydrofuran (DHMTHF), and dimethyltetrahydrofuran (DMTHF) can be also produced by reduction/hydrogenation processes [324–327]. The Piancatelli rearrangement of furylmethanols is a route towards cyclopentenones such as 4-hydroxy-4-(hydroxymethyl)cyclopentenone (HHCPN) or 3-(hydroxymethyl)cyclopentanone (HCPN) [168,328–329]. It is important to note that biocatalytic approaches have also been investigated for both the oxidation and reduction senses [172,330–331].

Because many reports have reviewed the formation of the HMF-derived scaffolds (Figure 4), this section will focus on transformations towards alternative fine chemicals, notably through cascade or multicomponent reactions.

**Scheme 97:**
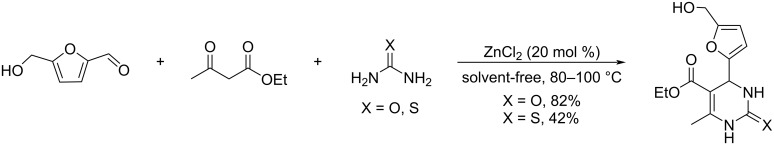
Biginelli reactions towards HMF-containing dihydropyrimidinones.

Combining atom-economic strategies with biobased platforms offers very interesting options for the innovative design of complex fine chemicals. Queneau and Popowycz investigated the reactivity of HMF in Biginelli reactions, preparing a series of functionalized dihydropyrimidinones in 30–86% yield. The reaction of HMF with ethyl acetoacetate (1 equiv) and urea or thiourea (1 equiv) at 80–100 °C using a catalytic loading of the Lewis acid ZnCl_2_ (20 mol %), provided respectively 82% and 42% of the corresponding dihydropyrimidinones (Scheme 97) [332]. Afradi et al. designed a nanocatalyst comprising aspartic acid‑loaded starch‑functionalized Mn/Fe/Ca ferrite magnetic nanoparticles for the efficient synthesis of dihydropyrimidine derivatives in solvent-free conditions [333].

**Scheme 98:**
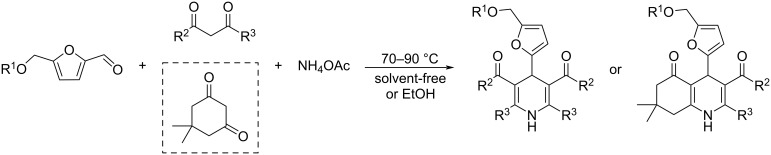
Hantzsch dihydropyridine synthesis involving HMF.

Queneau and Popowycz also recently reported the use of HMF in the Hantzsch dihydropyridine synthesis in the absence of any additional catalyst. The strategy was applied to a scope of β-dicarbonyl molecules in a three-component procedure leading to a series of symmetrical 1,4-dihydropyridines derived from 5-HMF and in a 4-component protocol which efficiently provided the corresponding unsymmetrical dihydropyridines under remarkably mild conditions (Scheme 98) [334].

**Scheme 99:**
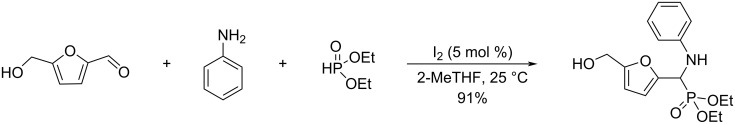
The Kabachnik–Fields reaction involving HMF.

Another multicomponent process involving HMF is the Kabachnik–Fields reaction which provides a variety of furan-based α-aminophosphonates in moderate to excellent yields (31–91%), using a catalytic quantity of iodine in the biobased solvent 2-methyltetrahydrofuran (2-MeTHF). A scope of aliphatic and aromatic amines and various dialkyl phosphonates were well tolerated in this reaction (Scheme 99) [335].

**Scheme 100:**
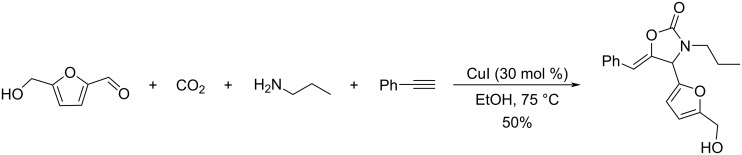
Construction of oxazolidinone from HMF.

In 2018, Chen and Wu reported a copper-catalyzed four-component reaction to build oxazolidinones from furfurals, CO_2_, terminal aromatic alkynes and primary aliphatic amines. A series of 1,3-oxazolidin-2-ones were obtained in 17–84% yield when CuI was used as catalyst. As an example, 50% yield of the 1,3-oxazolidin-2-one product was produced by the reaction of HMF, CO_2_, propylamine, and phenylacetylene (Scheme 100) [336].

**Scheme 101:**
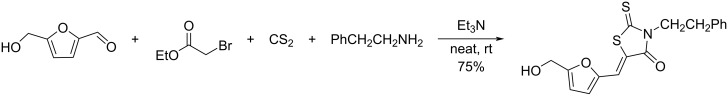
Construction of rhodamine-furan hybrids from HMF.

Rhodamine-furan hybrids were synthesized via one-pot stereoselective reaction in solvent-free conditions by Sabahi-Agabager. Starting from biobased furanic aldehydes (HMF, CMF, furfural), ethyl bromoacetate, carbon disulfide and phenethylamine, a 75% yield of rhodamine-furan hybrids was obtained at room temperature in the presence of Et_3_N (Scheme 101) [337].

**Scheme 102:**
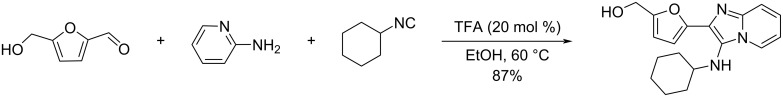
A Groebke–Blackburn–Bienaymé reaction involving HMF.

López and Porcal investigated the reactivity of HMF in the three-component Groebke–Blackburn–Bienaymé reaction. In the presence of 20 mol % of trifluoroacetic acid, the reaction of HMF with cyclic amidines (5- or 6-membered) and isocyanides afforded a library of new imidazoheterocycles. The best yield (87%) was obtained for the case of 2-aminopyridine and isocyanocyclohexane at 60 °C (Scheme 102) [338].

**Scheme 103:**
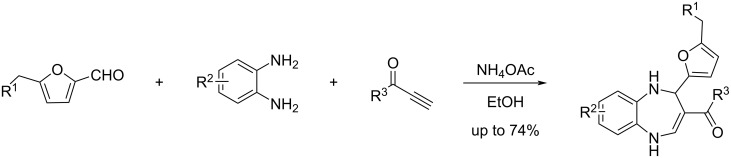
HMF-containing benzodiazepines by [4 + 2 + 1] cycloadditions.

A [4 + 2 + 1] cycloaddition strategy towards 1,5-benzodiazepines involving HMF was developed by the Queneau and Popowycz group with yields up to 74% by using ammonium acetate as both a mild catalyst and a source of nitrogen, in ethanol as a green solvent. The reaction scope was extended to various substituted diamines, alkynones/alkyl alkynoates, and furan aldehydes leading to a wide range of novel benzodiazepines (Scheme 103) [339].

**Scheme 104:**
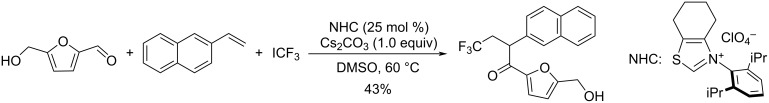
Synthesis of fluorinated analogues of α-aryl ketones.

HMF reactivity was investigated in several other MCRs. For example, Yang and Wu reported the use of *N*-heterocyclic carbene catalysis for the reaction of HMF with an alkene and a perfluoroalkyl reagent in the presence of Cs_2_CO_3_, providing access to the corresponding α-aryl-β-perfluoroalkyl products (Scheme 104). HMF led to a lower 43% yield compared to benzaldehyde (81%) and furfural (75%) [340].

**Scheme 105:**
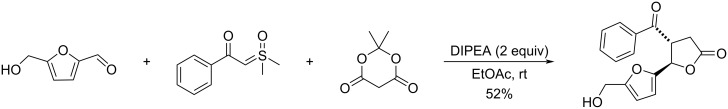
Synthesis of HMF derived disubstituted γ-butyrolactone.

The multicomponent reaction combining electron-rich aromatic aldehydes, sulfoxonium ylides and Meldrum’s acid can be performed in the presence of *N*,*N*-diisopropylethylamine to produce *trans*-β,γ-disubstituted-γ-butyrolactones in good yields (65–98%). In this process reported by Ma and Peng [341], HMF led to only a 52% yield of the target product due to competitive reaction of the free hydroxy group in HMF with Meldrum’s acid (Scheme 105).

**Scheme 106:**
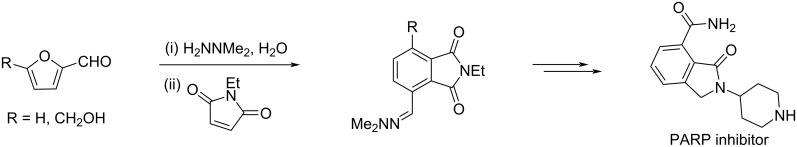
Functionalized aromatics from furfural and HMF.

A one-pot three-step cascade reaction involving HMF and other furanic aldehydes in the presence of dimethylhydrazine and phthalimide provides polysubstituted aromatics in water. The intermediate hydrazone formed from the aldehyde and dimethylhydrazine underwent in situ cycloaddition with the dienophile, before aromatization. This protocol afforded a range of polysubstituted aromatic compounds in high yields (up to 97%) and was employed to prepare a poly(ADP-ribose) polymerase (PARP) inhibitor (Scheme 106) [342].

**Scheme 107:**
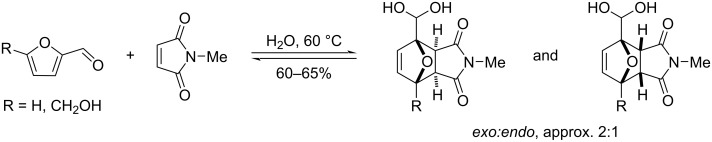
Diels–Alder adducts from HMF or furfural with *N*-methylmaleimide.

Several studies have concerned pericyclic cycloadditions involving HMF as diene component. For example, Bruijnincx reported a direct Diels–Alder reaction of HMF or furfural with *N*-methylmaleimide in aqueous medium to produce geminal diol products in 60 to 65% yield. Water is thought to pull the equilibrium toward the product side by chemically trapping the Diels–Alder adducts by hydratation (Scheme 107) [343].

**Scheme 108:**
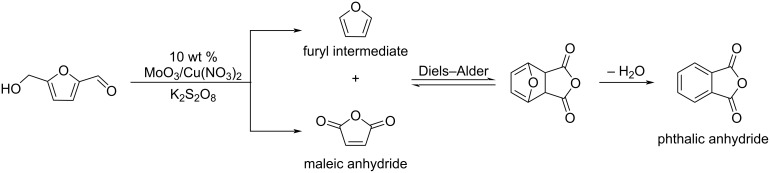
Pathway of the one-pot conversion of HMF into phthalic anhydride.

Sun reported the one-pot synthesis of phthalic anhydride by Diels–Alder reaction between HMF and maleic anhydride, promoted by a synergistic combination of MoO_3_ and Cu(NO_3_)_2_ catalysts. HMF can provide both partners of this Diels–Alder reaction, either by decarbonylation to an active furyl intermediate, and by oxidation to maleic anhydride. Subsequent dehydration of the DA adduct provided phthalic anhydride in 63% yield (Scheme 108) [344].

**Scheme 109:**
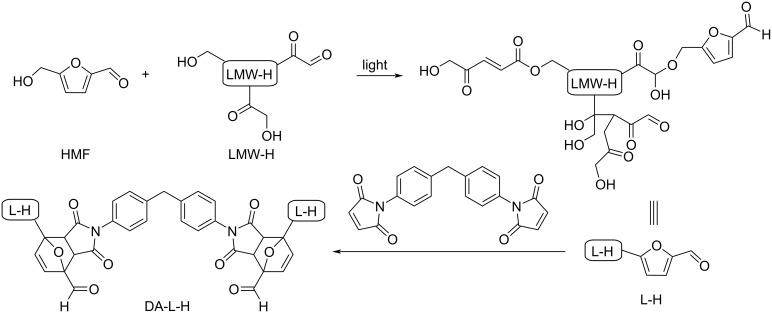
Photocatalyzed preparation of humins (L-H) from HMF mixed with spoiled HMF residues (LMW-H) and further cycloadditions with bismaleimide.

He and Guo reported the first photocatalyzed polymerization of HMF for the preparation of photo-generated humins (L-H) (Scheme 109). The L-H containing HMF and 5-hydroxy-4-ketopentenoic acid were generated by reacting HMF under illumination in presence of a low molecular weight humin (LMW-H) made from spoiled HMF residues. The resulting product allowed the preparation of furan-based polymers by Diels–Alder reaction with bismaleimidodiphenylmethane. A detailed physicochemical characterization indicated that the polymer DA-L-H had self-healing properties, and a recovery efficiency reaching 92.8% [345].

**Scheme 110:**
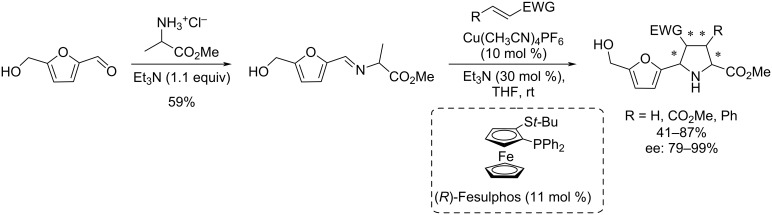
Asymmetric dipolar cycloadditions on HMF.

Reaction of HMF with alanine methyl ester hydrochloride in the presence of Et_3_N afforded HMF-derived iminoesters. By using the (*R*)-Fesulphos/Cu(CH_3_CN)_4_PF_6_ complex as a catalyst, the asymmetric 1,3-dipolar cycloaddition of the iminoester with activated alkenes provided enantiomerically enriched heterocyclic scaffolds in 41–87% yield and high enantioselectivity (79–99% ee) (Scheme 110) [346].

**Scheme 111:**
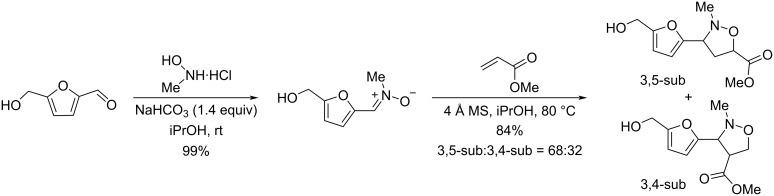
Dipolar cycloadditions of HMF based nitrones to 3,4- and 3,5-substituted isoxazolidines.

Queneau reported the synthesis of 3-furanylisoxazolidines by a dipolar cycloaddition of HMF-based nitrones with electron-deficient olefins in yields up to 84% for the stepwise reaction or 75% for the one-pot reaction. For example, *N*-methylhydroxylamine hydrochloride and HMF were condensed in isopropanol to form the corresponding nitrone, which underwent subsequently a cycloaddition with methyl acrylate in isopropanol to provide a regioisomeric mixture of 3,4-substituted and 3,5-substituted isoxazolidines in 84% yield (Scheme 111) [347].

**Scheme 112:**
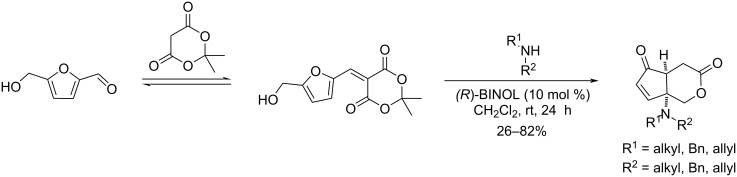
Production of δ-lactone-fused cyclopenten-2-ones from HMF.

Novel aminated lactone-fused cyclopenten-2-ones were prepared by reaction of the Knoevenagel product arising from Meldrum’s acid and HMF with a wide range of secondary amines. Through a furan ring-opening–cyclization–lactonization cascade process (closely related to the Piancatelli reaction), highly functionalized δ-lactone-fused cyclopenten-2-ones were formed in 26–82% yields under the promotion of (*R*)-BINOL acting as a weak Brønsted acid (Scheme 112) [348].

**Scheme 113:**
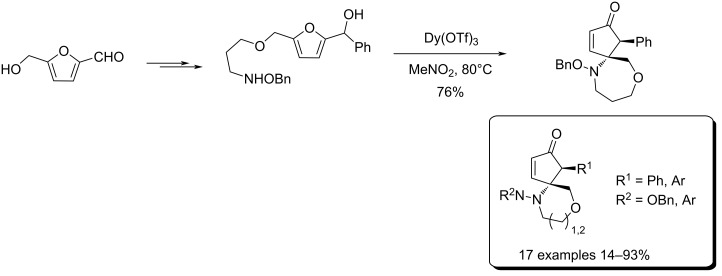
Aza-Piancatelli access to aza-spirocycles from HMF-derived intermediates.

Moebs-Sanchez and Popowycz recently reported a dysprosium triflate-catalyzed aza-Piancatelli reaction from HMF-derived intermediates forming cyclopentenone aza-spirocycles. A wide scope of novel 5,6- and 5,7-spirobicyclic scaffolds were prepared with yields up to 93% (Scheme 113) [349].

**Scheme 114:**
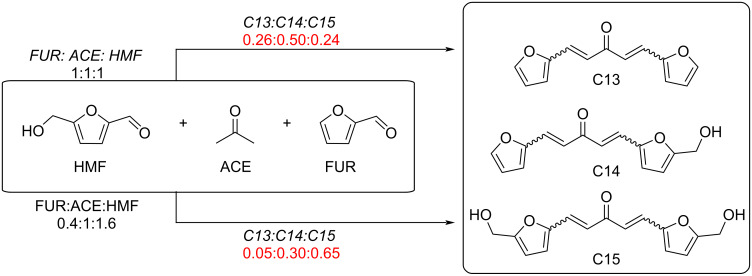
Cross-condensation of furfural, acetone and HMF into C_13_, C_14_ and C_15_ products.

The cross-aldol condensation of HMF, furfural and acetone can generate C_13_–C_15_ condensation products which are biofuels and polymer precursors [350]. Several solid metal catalysts and different ratios of starting materials were tested for comparing the reactivity of HMF and furfural in this process (Scheme 114).

**Scheme 115:**
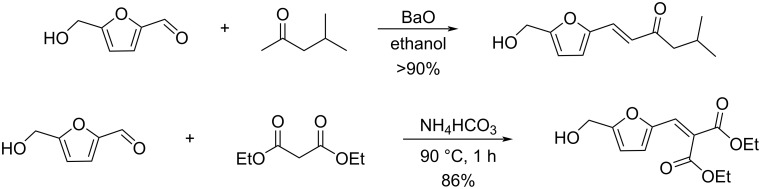
Base-catalyzed aldol condensation/dehydration sequences from HMF.

Ketones with enolizable α-H atoms can be coupled with HMF in alkaline medium to form new C–C bonds via cross-aldol condensation reaction. Examples include the use of alkyl ketones, cycloalkanones, and α,β-unsaturated ketones. Recently, Vigier used choline chloride to convert a carbohydrate feedstock into HMF which was then condensed with methyl isobutyl ketone (MIBK) in the presence of alkaline barium oxide as catalyst, affording 1-(5-(hydroxymethyl)furan-2-yl)-5-methylhex-1-en-3-one with yield over 90% [351]. Schijndel also reported a Knoevenagel condensation of furan aldehydes and diethyl malonate in the presence of ammonium bicarbonate giving a 86% yield of the HMF-derived condensation product [352] (Scheme 115).

**Scheme 116:**
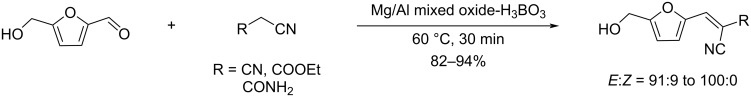
Condensation of HMF and active methylene nitrile.

Mancipe and Luque reported the use of a boric acid deposited on hydrotalcite catalyst for the Knoevenagel condensation of HMF with active methylene compounds to afford a series of HMF derivatives containing an acrylonitrile moiety. The acid–base catalytic sites accelerate the reaction leading to high yields (82–94%) and high (*E*)-isomer selectivity (91:9 to 100:0) of the condensation products (Scheme 116) [353].

**Scheme 117:**
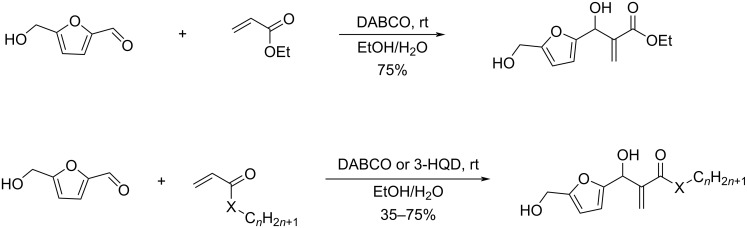
MBH reactions involving HMF.

Another carbon–carbon-bond formation involving the aldehyde group of HMF is the Morita–Baylis–Hillman (MBH) reaction. Queneau showed that the reaction with acrylates in the presence of DABCO can take place in a combination of biobased solvents such as EtOH/H_2_O 1:1 (v/v) to replace traditional solvents, with yields up to 90% after 24 h [354]. The strategy was later applied to the preparation of original biobased amphiphiles from hydrophobic alkenes. These amphiphiles were shown to emulsify both polar and apolar oils, providing W/O and O/W emulsions, suggesting that they could be used as relevant alternatives to traditional petroleum-based polyethoxylated surfactants (Scheme 117) [355].

**Scheme 118:**
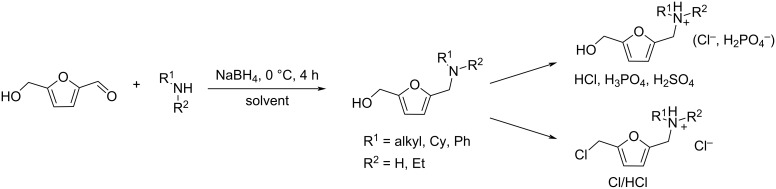
Synthesis of HMF-derived ionic liquids.

Finally, several strategies based on the reductive amination of HMF have been reported. For example, Ananikov synthesized a series of multifunctional protic ammonium ionic liquids with various inorganic anions by reductive amination of HMF with various amines (Scheme 118). The salts were studied with respect to their solvent properties. Interestingly, the ionic liquids containing sulfate anions have the ability to dissolve cellulose. Regarding the biological properties, the introduction of a chlorine substituent into the side chain of the furfural unit is beneficial to the bactericidal effect. A study on physicochemical properties indicated that most of these ionic liquids were stable when stored for more than six months [356].

**Scheme 119:**
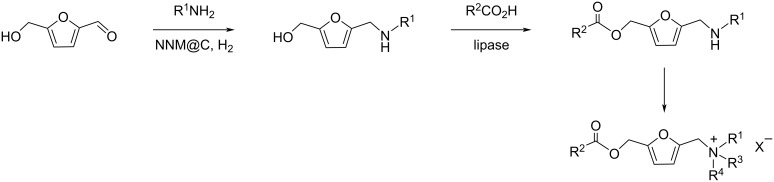
Reductive amination/enzymatic acylation sequence towards HMF-based surfactants.

Heterogeneous monometallic (Ni, Co, Ru, Pd, Pt, Rh) and bimetallic (Ni/Cu, Ni/Mn) catalysts have been shown to be efficient for the reductive amination of HMF [357–358]. A recent application towards surfactant precursors has been reported by Iborra and Corma, using a non-noble metal (NNM) catalyst based on monodisperse cobalt nanoparticles coated with a few layers of carbon (NNM@C). Subsequent lipase-catalyzed acylation using carboxylic acids afforded the aminoesters, one step away from cationic surfactants by amine quaternization (Scheme 119) [359]. This strategy provided additional methods towards biobased furanic surfactants for which two reviews were reported in 2022 [173,360].

#### 5-(Chloromethyl)furfural (CMF)

**Scheme 120:**
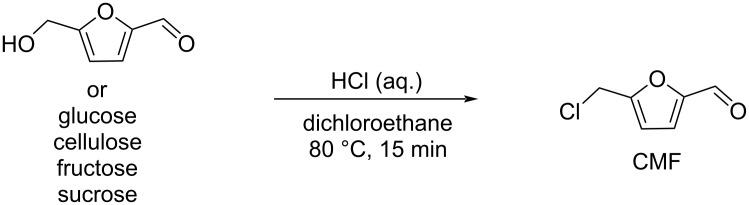
The formation of 5-chloromethylfurfural (CMF).

5-(Chloromethyl)furfural (CMF) can be obtained from raw biomass such as bagasse, cassava wastes, corn stover, bamboo powder or a variety of pure carbohydrates, such as glucose, sucrose, fructose, cellulose. CMF has gained popularity thanks to the pioneering work and accounts reported by Mascal, showing the potential of this alternative platform for monomers, biofuels and specialty chemicals [361–362]. Several other groups have contributed to the topic, and a few more in-depth perspectives on the synthesis and applications of CMF and other 5-(halomethyl)furfurals have been reported [363–364]. A popular system to access CMF is the use of HCl as chlorinating agent in dichloroethane, which can be achieved from HMF (Scheme 120), but also from glucose, sucrose or cellulose. Compared to HMF, the main differences are the higher stability of CMF under acidic conditions and its easier extractability from the medium due to a higher lipophilicity. In the following we give a brief and non-comprehensive overview of CMF downstream chemistry illustrating its usefulness towards fine chemicals.

**Scheme 121:**
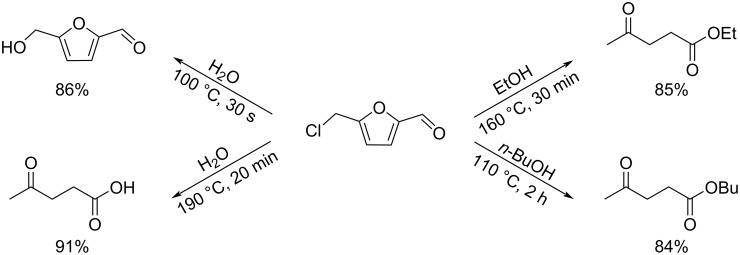
Conversion of CMF to HMF, levulinic acid, and alkyl levulinates.

CMF can be used as a stable intermediate for the production of HMF, levulinic acid and alkyl levulinate (EL and BL) via hydrolysis or alcoholysis (Scheme 121) [365]. HMF is obtained in 86% yield from CMF in boiling water for only 30 seconds, while heating CMF in dilute hydrochloric acid at 150 °C for 5 h or without acid at 190 °C leads to levulinic in high yield. If alcohols are added, high yields of alkyl levulinates are directly obtained.

**Scheme 122:**
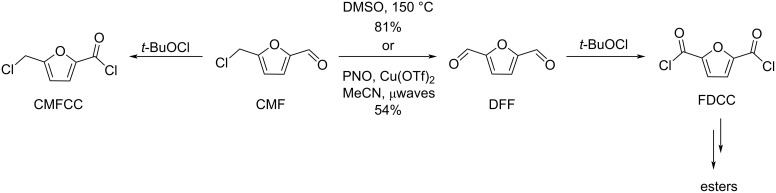
Conversion of CMF to CMFCC and FDCC.

2,5-Diformylfuran (DFF) can be obtained in 81% yield from CMF in DMSO, as proposed by Estrine and Le Bras [366] or in 54% yield over the combined pyridine *N*-oxide and Cu(OTf)_2_ catalyst when using acetonitrile under microwave irradiation as reported by Afonso [367]. CMF can be also directly oxidized to 5-(chloromethyl)furan-2-carbonyl chloride (CMFCC) or converted to furan-2,5-dicarbonyl chloride (FDCC) via the formation of intermediate 2,5-diformylfuran (DFF) after reaction with *tert*-butyl hypochlorite (*t*-BuOCl) (Scheme 122) [368]. Being conveniently derivatizable acid chlorides, CMFCC and FDCC are highly versatile intermediates for the production of furans presenting a carboxylic acid function or other downstream chemical precursors of polymers and biofuels.

**Scheme 123:**
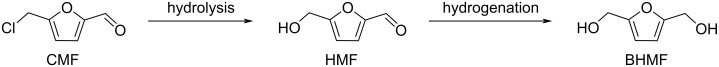
Conversion of CMF to BHMF.

Suh has shown that CMF can be derivatized towards BHMF, using a stepwise hydrolysis and hydrogenation via HMF over mesoporous Cu/Al_2_O_3_ (meso-CuA-kg) under H_2_ pressure [369]. Recently, Zeng reported the one-pot consecutive hydrolysis and hydrogenation of CMF to BHMF in aqueous phase in 69% overall yield. Under the synergistic effect of Ru and Cu_2_O species, 91% BHMF was obtained directly from CMF over Ru/CuO_x_ as catalyst at 60 °C and 4 MPa H_2_ without isolation of the intermediate HMF [370] (Scheme 123).

**Scheme 124:**
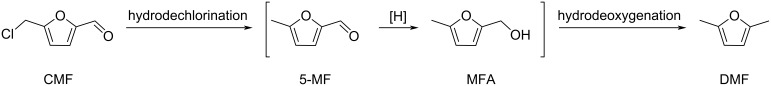
Conversion of CMF to DMF.

Zeng has reported the reduction of CMF to 2,5-dimethylfuran (DMF) under mild conditions using a Cl-modified Pd catalysts (Pd/CNTs) and 2 MPa of H_2_ affording 92% yield after 15 min at 30 °C (Scheme 124) [371].

**Scheme 125:**
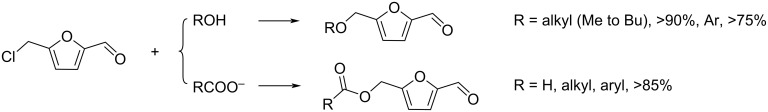
CMF chlorine atom substitutions toward HMF ethers and esters.

The substitution of the chlorine atom of CMF by nucleophiles produces either ethers [372–373] or esters [374] from alcohols or carboxylic acids, respectively, allowing the preparation of a library of functionalized furanic aldehydes (Scheme 125). For example, Dutta [372] and Sudarsanam [374] recently synthesized a series of alkyl/aryl-substituted HMF esters in high yields (>85%) by reacting CMF and various carboxylic acids in the presence of a triethylammonium salt under solvent-free conditions (80 °C, 1.5 h). The two-step process from cellulose allowed the preparation of 5-(acetoxymethyl)furfural in an overall yield of nearly 65%.

**Scheme 126:**
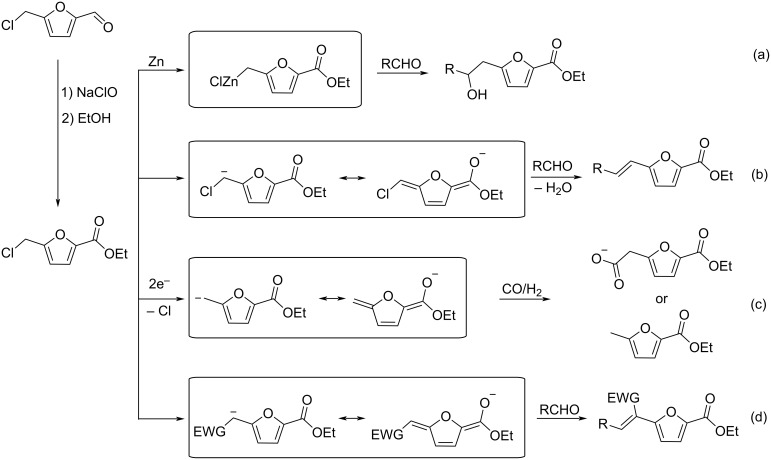
Introduction of carbon nucleophiles in CMF.

Carbon nucleophiles can also substitute the chlorine atom of CMF. For example, Zn metal insertion into the C–Cl bond of ethyl 5-(chloromethyl)furan-2-carboxylate can produce organozinc furoate esters able to react with a range of aldehydes (Scheme 126, reaction a) [375]. Alternatively, in a Reformatsky-type process, deprotonation of the 5-(chloromethyl)furoate esters and reaction with aldehydes followed by dehydration results in the formation conjugated alkenyl furoates which have applications in dyes or epoxy resin fields (Scheme 126, reaction b) [376]. The furylogous enolate anion resulting from the electrochemical reductive cleavage of ethyl 5-(chloromethyl)furan-2-carboxylate derived from CMF can react with carbon dioxide to yield 5-(carboxymethyl)furan-2-carboxylate, a useful intermediate towards the fuel additive 5-methylfuran-2-carboxylate commercially referred to as "Ethyl 408" (Scheme 126, reaction c) [377]. Similarly, furoate esters derived from CMF can undergo nucleophilic substitution with cyanide to produce furylogous cyanoacetic esters, employed for the design of biobased dyes (Scheme 126, reaction d) [378].

**Scheme 127:**
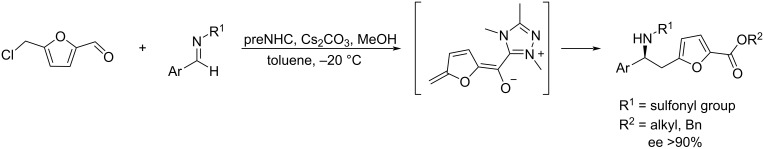
NHC-catalyzed remote enantioselective Mannich-type reactions of CMF.

Gao and Ye reported the bifunctional-NHC-catalyzed enantioselective Mannich-type reaction involving CMF (Scheme 127). The products were obtained in high yields and enantioselectivities with excellent functional group tolerance [379].

**Scheme 128:**
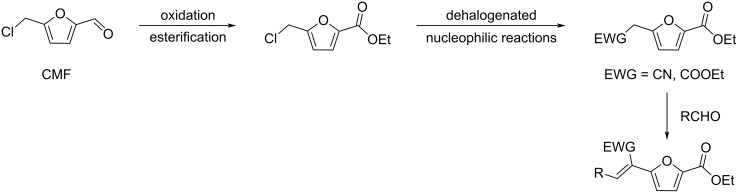
Conversion of CMF to promising biomass-derived dyes.

Biobased dyes have been obtained by introducing strong chromophores on the furanoate scaffold through the Knoevenagel reaction of CMF with aldehydes (Scheme 128). The reaction of furylogous malonate or furylogous cyanoacetate and biomass-derived aldehydes produced brightly colored products from the yellow to red region of the spectrum, exhibiting good color properties and color fastness on selected fabrics [378].

**Scheme 129:**
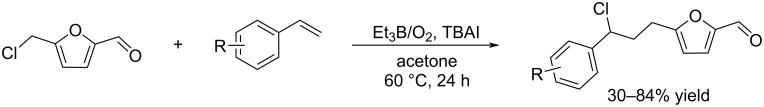
Radical transformation of CMF with styrenes.

Rehbein and Brasholz have reported the first radical transformation at the benzylic chloromethyl group of CMF. A series of atom transfer radical addition products were obtained by reacting CMF with styrenes under triethylborane (Et_3_B)/O_2_ system (Scheme 129) [380].

**Scheme 130:**
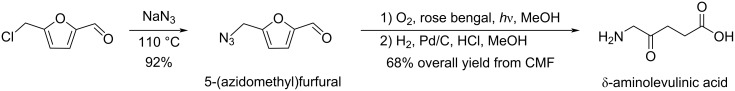
Synthesis of natural herbicide δ-aminolevulinic acid from CMF.

CMF can also react with sodium azide as applied in a synthesis of δ-aminolevulinic acid, an important agricultural and pharmaceutical intermediate. The reaction of CMF and NaN_3_ led to the formation of 5-(azidomethyl)furfural. Subsequent photooxidation by irradiation with singlet oxygen, ring opening of the furan and subsequent catalytic hydrogenation resulted in the formation of δ-aminolevulinic acid in 68% yield (Scheme 130) [362].

**Scheme 131:**
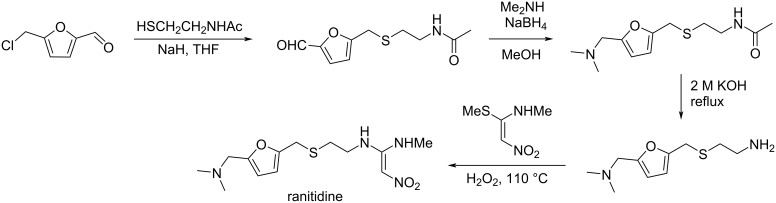
Four step synthesis of the drug ranitidine from CMF.

The substitution of the chlorine atom of CMF by a sulfur atom has also been reported like in a four-step synthesis of ranitidine, a histamine H_2_ receptor antagonists used to treat conditions related to excess stomach acid production. Once the sulfide bond was formed, the (dimethylamino)methyl group was introduced through reductive amination of the aldehyde group (Scheme 131) [381]. Removal of the acetyl group by treatment with KOH led to the previously reported amino intermediate [382] which afforded ranitidine by addition–elimination reaction on 1-methylthio-1-methylamino-2-nitroethylene in overall 68% yield from CMF.

#### 1-Hydroxy-2,5-hexanedione (HHD) and 2,5-hexanedione (HXD)

**Scheme 132:**
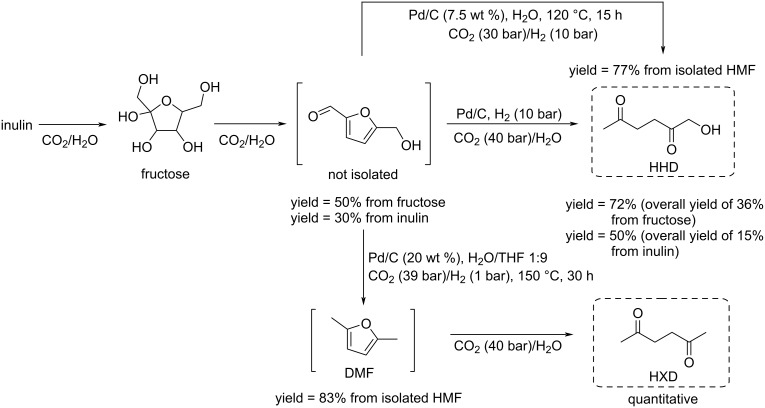
Pd/CO_2_ cooperative catalysis for the production of HHD and HXD.

Linear diketones such as 1-hydroxyhexane-2,5-dione (HHD) or 2,5-hexanedione (HXD) are promising next-generation biomass-derived platform molecules [383–384]. Many conditions have been developed for the catalytic hydrogenation of HMF to HHD using various metals (Pd, Ir, Ru, Rh, Au, Ni-based). For example, Jérome and De Campo reported the one-pot production of HHD directly from carbohydrates (fructose and inulin) through a bifunctional catalytic process using the dual Pd/C/H_2_-CO_2_/H_2_O system, which afforded HHD in 36% and 15% yields, respectively. Pd/C-catalyzed hydrogenation of HMF provided HHD with up to 77% yield. 2,5-Hexanedione (HXD) could be obtained through another route in high yield via 2,5-dimethylfuran (DMF) (Scheme 132) [385]. When using a Pd/C and Amberlyst-15 system, fructose and inulin were found to be converted into HHD in yields of 55% and 27%, respectively [386].

**Scheme 133:**
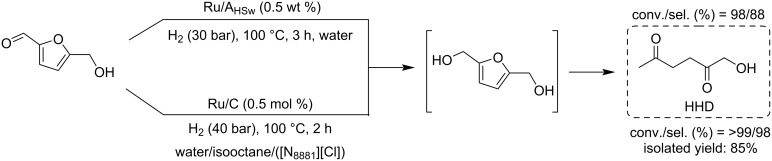
Different ruthenium (Ru) catalysts for the ring-opening of 5-HMF to HHD.

Signoretto reported the hydrogenation of HMF to HHD using a Ru catalyst supported on biochar obtained by pyrolysis of hazelnut shells (Ru/A_HSw_), a sustainable and inexpensive catalyst. An 88% selectivity for HHD was observed with full conversion of HMF at 30 atm of H_2_ (Scheme 133) [387]. In 2023, Selva published a method for the preparation of HHD from HMF by Ru/C catalysis in an ionic liquid-assisted three-phase system consisting of water, isooctane and methyltrioctylammonium chloride ([N_8881_][Cl]), providing HDD with 98% selectivity and 85% isolated yield with full conversion of HMF (>99%). The separation of the product and catalyst (Ru/C) could be easily achieved by adjusting the ratio of the multiphase components (Scheme 133) [388].

**Scheme 134:**
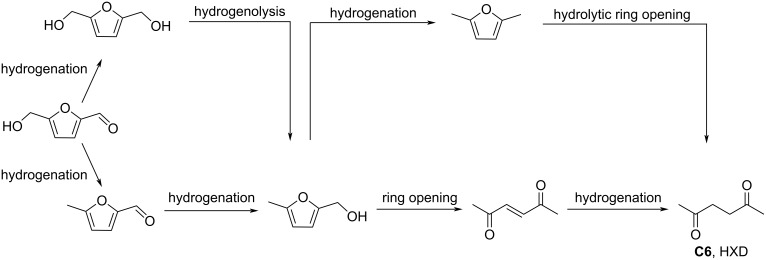
Proposed pathways for preparing HXD from HMF.

2,5-Hexanedione (HXD) could be directly obtained from HMF, DMF or cellulose via acid-catalyzed hydrolysis and metal-catalyzed hydrogenolysis. An iodine-modified Pd catalyst PdI/Al_2_O_3_ promoted the hydrogenative ring opening of HMF in water at 110 °C under 4.0 MPa H_2_, providing HXD in 93% yield [389], while a Pd/Ti_3_AlC_2_ catalyst promoted the same conversion in water and 4.0 MPa H_2_ at only 90 °C [390]. The heterogeneous catalyst cobalt disulfide (CoS_2_) with imperfect spherical (A-CoS_2_) showed high activity in the hydrogenolysis of HMF to HXD with a yield of 81.5 wt % at 200 °C under H_2_ pressure (2 MPa) in MeOH. When applied directly to cellulose, an 11.8 wt % yield of HXD was obtained under these conditions [391]. Various homogeneous acidic catalysts such as Al_2_(SO_4_)_3_, AlCl_3_, Fe_2_(SO_4_)_3_, FeCl_3_, SnCl_4_, H_2_SO_4_ and supported noble-metal catalysts (Pd/C, Pt/C, and Ru/C) were tested in the one-pot catalytic conversion of cellulose to HXD developed by Liu and Shu [392]. The combination of Al_2_(SO_4_)_3_ with Pd/C exhibited the highest catalytic activity, avoiding excessive hydrogenation of furan intermediates, with an 80% yield of HXD obtained in H_2_O/THF at 190 °C and H_2_ (2 MPa). Glucose and sucrose gave 91% and 83% yield of HXD at 200 °C and H_2_ (2 MPa), respectively. A proposed pathway for the formation of HXD from 5-HMF is shown in Scheme 134.

**Scheme 135:**
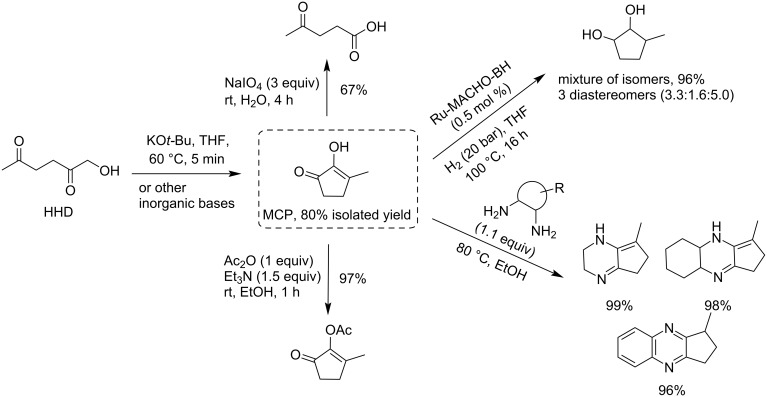
MCP formation and uses.

**Conversions of HHD:** HHD can be further converted into 2-hydroxy-3-methylcyclopent-2-enone (MCP) via intramolecular aldol reactions. In 2018, de Vries developed an efficient base-catalyzed process for the conversion of HHD into MCP. MCP is a hub chemical which can be further transformed into levulinic acid, enol acetate, diol and *N*-heterocyclic compounds through oxidative ring cleavage, acetylation, hydrogenation, and condensation reactions, respectively (Scheme 135) [393].

**Scheme 136:**
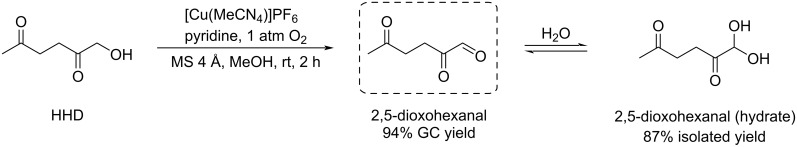
Cu(I)-catalyzed highly selective oxidation of HHD to 2,5-dioxohexanal.

In 2022, de Vries reported the selective Cu(I)-catalyzed oxidation of α-hydroxy ketones to α-keto aldehydes. A 94% GC yield of 2,5-dioxohexanal was obtained from HHD under oxygen atmosphere in the presence of [Cu(MeCN)_4_]PF_6_ (5 mol %), pyridine (10 mol %) and 4 Å molecular sieves (MS). The aqueous work-up of the reaction resulted in the formation of the α-keto aldehyde hydrate in 87% isolated yield (Scheme 136) [394].

**Scheme 137:**
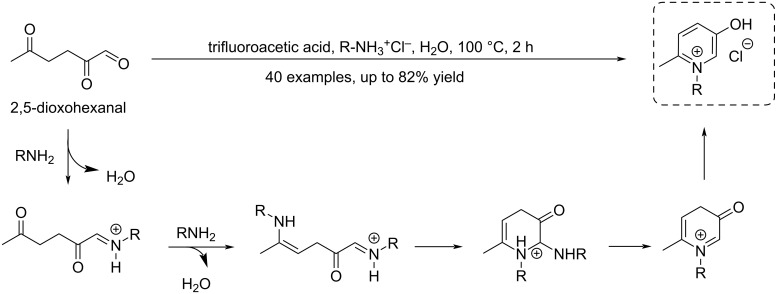
Synthesis of *N*‑substituted 3‑hydroxypyridinium salts from 2,5-dioxohexanal.

This HHD-2,5-dioxohexanal route was also used by the same team to access a wide range of *N*-alkyl and *N*-aryl-3-hydroxypyridinium salts with various functional groups in good yields (up to 82% yield) in the presence of trifluoroacetic acid (Scheme 137). The salts can be used as intermediates in the synthesis of some nitrogen-containing scaffolds [395].

**Scheme 138:**
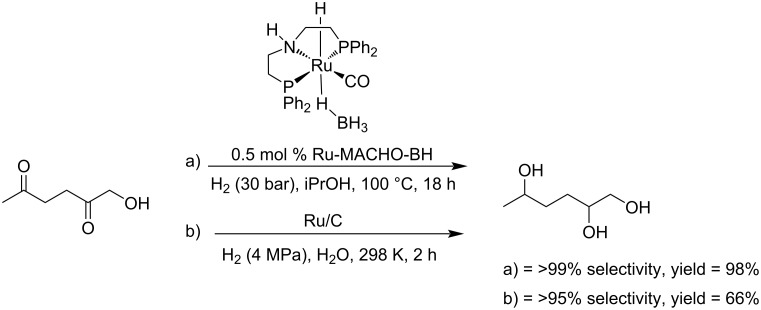
Ru catalyzed hydrogenations of HHD to 1,2,5-hexanetriol (a) see ref. [396]; b) see ref. [397]).

HHD total hydrogenation to 1,2,5-hexanetriol was achieved by de Vries using a Ru-MACHO-BH catalyst (0.5 mol %) under 30 atm H_2_ at 100 °C in 18 hours in isopropanol as solvent with nearly quantitative yield (Scheme 138A) [396]. Alternatively, Yang proposed a Ru/C catalyst for the same reaction, giving 1,2,5-hexanetriol in 66% yield with 95% selectivity and 99% conversion of HHD under mild reaction conditions (Scheme 138B) [397].

**Scheme 139:**
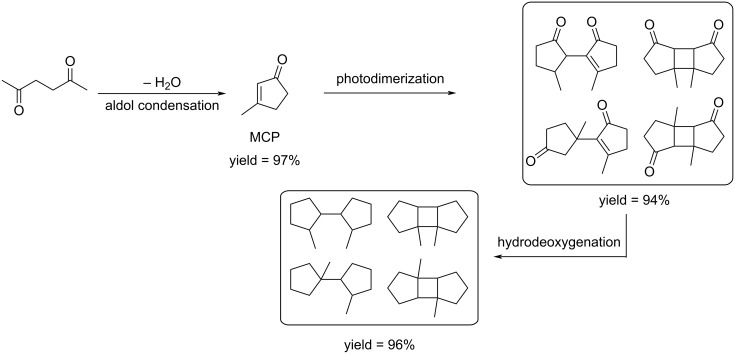
Aviation fuel range quadricyclanes produced by HXD.

**Conversions of HXD:** 3-Methylcyclopent-2-enone (MCP) can be obtained from HXD in 97% yield by cyclization promoted by sodium carbonate in a toluene/H_2_O biphasic system. Subsequent dimerization of MCP under ultraviolet irradiation provided polycyclic C_12_ diketones which were hydrodeoxygenated under acidic zeolite H-Y and commercial Ru/C catalysis, leading to a high-density C_12_ polycycloalkane mixture with low freezing point consistent with the usage as aviation fuel (Scheme 139) [398].

**Scheme 140:**
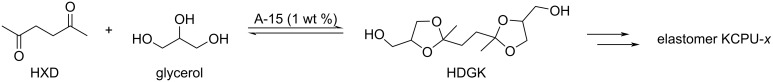
Synthesis of HDGK from HXD and glycerol as a chain extender.

Yan and Chen synthesized the 2,5-hexadione glycerol ketal (HDGK) by reaction of HXD and glycerol, and used it as a chain extender and crosslinking agent to prepare KCPU-*x*, a dynamic covalent cross-linked polyurethane elastomer (Scheme 140) [399]. The incorporation of a cyclic ketal structure improved the stability, dielectric properties, and healing efficiency of these polyurethane elastomers.

**Scheme 141:**
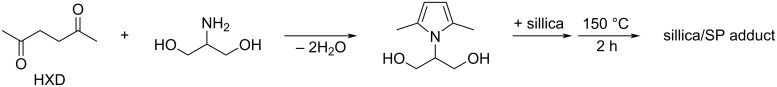
Synthesis of serinol pyrrole from HXD and serinol.

HXD can react with serinol to form 2-(2,5-dimethyl-1*H*-pyrrol-1-yl)-1,3-propanediol (serinol pyrrole, SP) which was used as a high efficiency coupling agent between silica and unsaturated polymer chains in elastomer composites for tires, applicable on the industrial scale (Scheme 141) [400].

**Scheme 142:**
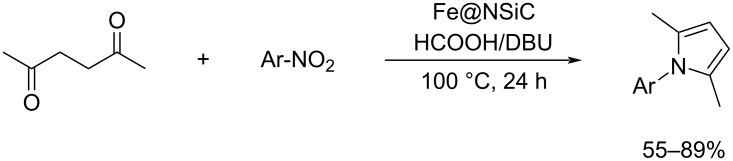
Synthesis of pyrroles from HXD and nitroarenes.

A series of pyrroles, among which some are important intermediates in drug design, has been obtained in 55–89% yields by the cascade reaction of nitroarenes with HXD over a carbon-based iron heterogeneous catalyst (Fe@NSiC) using HCOOH/DBU as reducing agent (Scheme 142) [401].

**Scheme 143:**
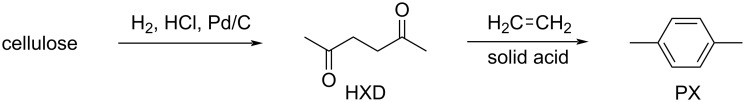
Two-step production of PX from cellulose *via* HXD.

Efficient upgrading of cellulose into *p*-xylene (PX) was reported through intermediate HXD by Chu et al. [402]. Following a new two-step route (Scheme 143), an 64% overall yield of HXD and DMF was obtained from cellulose, and PX was obtained in 55% yield from this mixture using a solid acid like SnPO-1.75 as catalyst at 275 °C and 2 MPa H_2_.

#### 3-(Hydroxymethyl)cyclopentanone (HCPN)

**Scheme 144:**
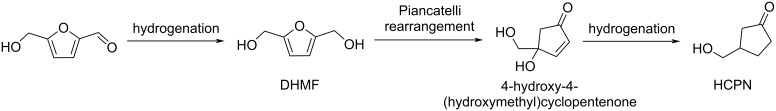
Preparation of HCPN from HMF via hydrogenation and ring rearrangement.

3-(Hydroxymethyl)cyclopentanone (HCPN) can be obtained from HMF, usually involving the hydrogenation of HMF to 2,5-dihydroxymethylfurfural (DHMF), and subsequent Piancatelli rearrangement to 4-hydroxy-4-(hydroxymethyl)cyclopentenone followed by hydrogenation to HCPN (Scheme 144) [168,328].

The first reported conversion of HMF to HCPN in 2014 used metal oxide-supported Au nanoparticles as catalysts, with Au/Nb_2_O_5_ providing the best yield (86%) in the presence of H_2_ (8 MPa) at 140 °C [403]. Since then, several other supports such as SiO_2_, Al_2_O_3,_ Al_2_O_3_ pyrochlore composites, MOFs, and DMC (double-metal cyanide) have been used for Pt, Pb, Ir catalysts and a palladium bifunctional catalysis method has been reported [404–405]. In 2016, Rosseinsky reported a first non-noble-metal-catalyst derived from Ni/Al layered double hydroxides (Ni-on-Al_2_O_3_), that afforded an 81% yield of HCPN from HMF [406]. Subsequently, other non-noble-metal-based bimetallic catalysts were applied for this transformation such as Cu-Al_2_O_3_, Ni-Cu/MOF and Ni-Fe/Al_2_O_3_ [407–410].

**Scheme 145:**
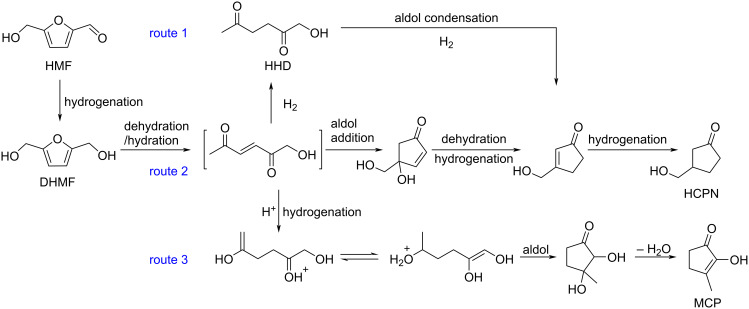
Suggested pathways from HMF to HCPN.

While route 1 and route 2 mechanisms shown in Scheme 145 were previously proposed for the conversion of HMF to HCPN, Deng and Fu expressed doubts about the involvement of intermediate 1-hydroxy-2,5-hexanedione (HHD). They considered that Brønsted acid catalysis promotes the formation of HHD and subsequently 3-methylcyclopenten-2-ol-1-one (MCP) instead of HCPN (Scheme 145, route 3), while Lewis acids facilitate the production of a precursor of HHD, then generating HCPN [411].

**Scheme 146:**
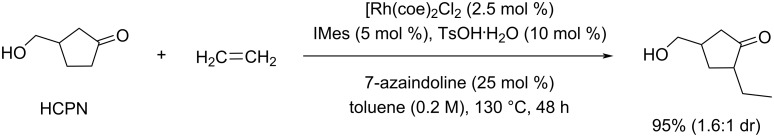
α-Alkylation of HCPN with ethylene gas.

HCPN can undergo all typical reactions of ketones, and only two have been selected here, an alkylation and a reductive amination. In 2014, Dong reported the regioselective α-alkylation of HCPN using ethylene as the alkylating agent in the presence of chlorobis(cyclooctene)rhodium(I) dimer [Rh(coe)_2_Cl]_2_, 1,3-bis(2,4,6-trimethylphenyl)imidazole-2-ylidene (IMes), *p*-toluenesulfonic acid monohydrate (TsOH·H_2_O), and 7-azaindoline, leading to α-ethylated HCPN in 95% isolated yield (1.6:1 dr) (Scheme 146) [412].

**Scheme 147:**
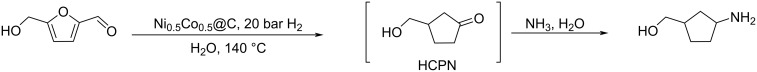
Synthesis of 3-(hydroxymethyl)cyclopentylamine from HMF via reductive amination of HCPN.

An efficient one-pot cascade conversion of HMF promoted by the Ni_0.5_Co_0.5_@C catalytic system formed 3-(hydroxymethyl)cyclopentylamine through reductive amination of HCPN with ammonia at 140 °C and 20 bar H_2_ for 8 h, with 97% selectivity and full conversion of HCPN (Scheme 147) [413].

#### Levoglucosenone (LGO) and dihydrolevoglucosenone (H_2_LGO or Cyrene^®^)

**Scheme 148:**
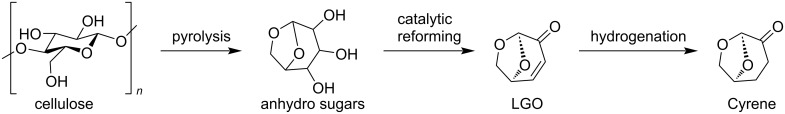
Production of LGO and Cyrene^®^ from biomass.

Levoglucosenone (LGO) and its fully hydrogenated derivative, dihydrolevoglucosenone (H₂LGO), commercially known under the trademark Cyrene^®^, are two highly promising biobased C_6_ platform molecules. Derived from renewable cellulosic resources, these compounds offer versatile and sustainable scaffolds for the synthesis of a wide range of value-added chemicals, materials, and pharmaceuticals (Scheme 148). LGO, which arises from cellulose by pyrolysis [414–416], or levoglucosan [417] exhibits a bicyclic structure possessing three reactive functions, namely an acetal, a ketone and its conjugated double bond. The selective hydrogenation of the double bond can produce the corresponding saturated ketone dihydrolevoglucosenone (H_2_LGO, Cyrene^®^) [418], which has intensively been studied as a sustainable alternative to classical aprotic dipolar solvents [419–422].

Both LGO and Cyrene^®^ molecules offer a wide range of transformations towards intermediates enabling innovative design in fine chemistry and polymer science [423–425]. The following sections highlight the versatility and practical utility of LGO and Cyrene^®^ through selected recent examples, demonstrating their potential as valuable intermediates in sustainable synthesis.

**Scheme 149:**
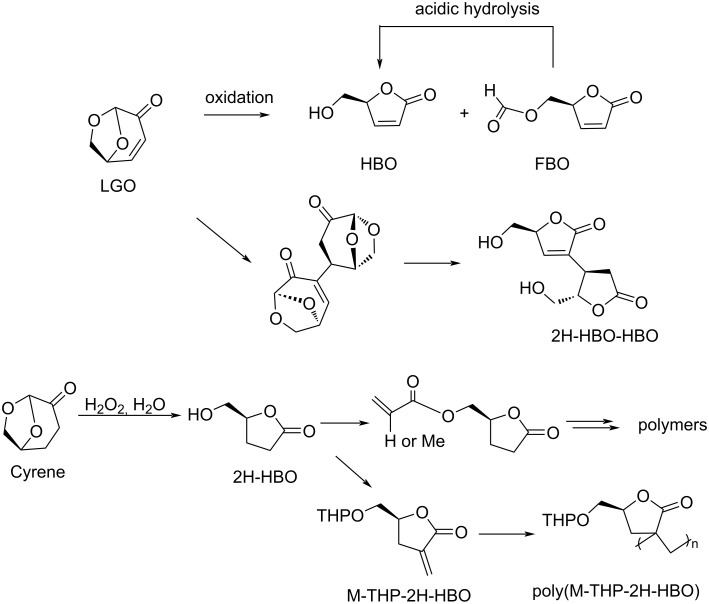
Synthesis of HBO from LGO and other applications.

LGO can be oxidized to (*S*)-γ-hydroxymethyl-α,β-butenolide (HBO) and its formate ester under Baeyer–Villiger conditions (Scheme 149). Hydrolysis of the formate FBO allows a complete transformation of LGO to HBO. When applied to Cyrene^®^, the reaction leads to the corresponding hydrogenated (*S*)-γ-(hydroxymethyl)butyrolactone (2H-HBO) [426]. The Allais group has reported yields up to 72% on a kilogram scale synthesis, employing aqueous hydrogen peroxide as both the solvent and the oxidizing agent [427]. The sterically hindered bicyclic diol 2H-HBO-HBO can be obtained from LGO through a sustainable two-step process involving LGO homocoupling followed by Baeyer–Villiger oxidation [428]. Several polymers have been obtained after acryloylation or methacryloylation of the OH group [429]. Starting from Cyrene^®^, Miller and Allais designed a methylated derivative of HBO. After protection of the hydroxy group as a tetrahydropyranyl (THP) acetal giving the intermediate 2-THP-2H-HBO, the α-methylenated derivative (M-THP-2H-HBO) was obtained by reaction of its enolate with paraformaldehyde. This latter was found to slowly oligomerize [430].

**Scheme 150:**
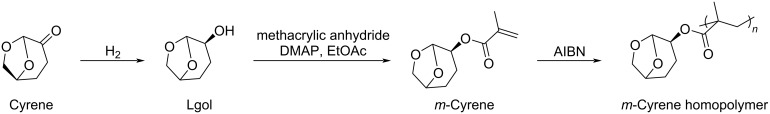
Construction of *m*-Cyrene^®^ homopolymer.

If the hydrogenation of LGO is continued further after formation of Cyrene^®^, the ketone is reduced and levoglucosanol (Lgol) is formed. For example, in the presence of Pd/C and HCOOH as hydrogen source, the Lgol yield can reach 95% at 180 °C [431]. A methacrylic monomer (*m*-Cyrene^®^) formed from Lgol was found to undergo rapid polymerization or copolymerization in the presence of AIBN and in Cyrene^®^ as solvent (Scheme 150) [429].

**Scheme 151:**
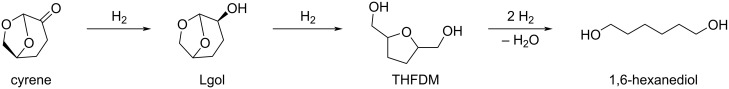
Conversion of Cyrene^®^ to THFDM and 1,6-hexanediol.

Tetrahydrofurandimethanol (THFDM) and 1,6-hexanediol could be prepared from Lgol via complete hydrogenation/hydrogenolysis [432]. Huber reported a conversion of Lgol to THFDM with 78% overall selectivity to 1,6-hexanediol at 150 °C using a bifunctional Pd/SiO_2_/Al_2_O_3_ catalyst (Scheme 151) [433].

**Scheme 152:**
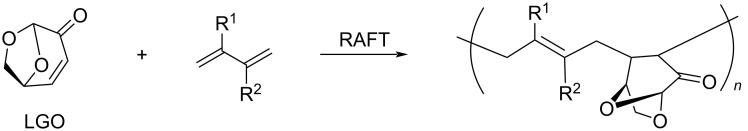
RAFT co-polymerization of LGO and butadienes.

A recent example of reversible addition–fragmentation chain transfer (RAFT) copolymerization of LGO and butadiene or 2- or 3-methyl derivatives was reported by the Choi group (Scheme 152). Conversions of LGO and butadiene up to 80% were observed, leading to polymers having a high degree of alternation and exhibiting excellent thermal stability [434].

**Scheme 153:**
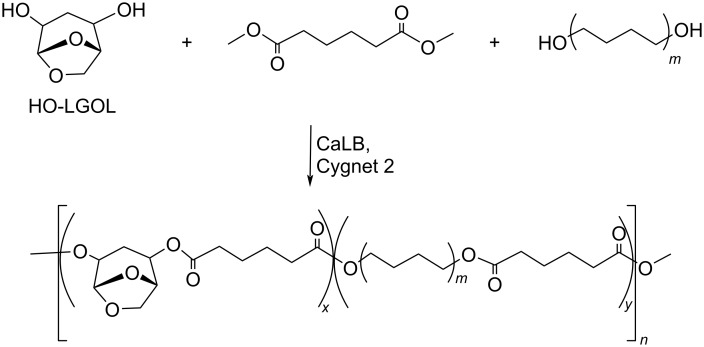
Polycondensation of HO-LGOL and diols with dimethyl adipate.

Another interesting example of the use of LGO in polymer science was recently reported by Warne and Pellis, who used the diol obtained by hydration and reduction of LGO in combination with butane-1,4-diol for polycondensation with dimethyl adipate in the green solvent dioxolane Cygnet 2 under biocatalytic conditions (*Candida antartica* lipase B CaLB) (Scheme 153). Longer diols such as 1,8-octanediol or 1,12-dodecanediol were also used [435].

**Scheme 154:**
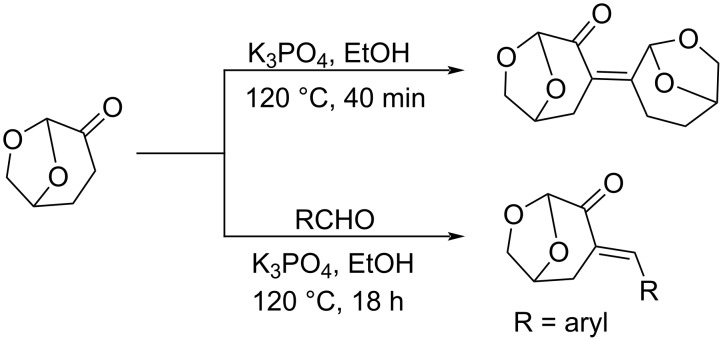
Self-condensation of Cyrene^®^ and Claisen–Schmidt reactions.

In the presence of a catalytic quantity of K_3_PO_4,_ the self-condensation of Cyrene^®^ gave the corresponding product in 81.3% yield at 120 °C for 40 min. The Claisen–Schmidt reaction of Cyrene^®^ with aromatic and heteroaromatic aldehydes offered a wide range of yields, up to 95% at 120 °C after 18 h (Scheme 154) [436].

**Scheme 155:**
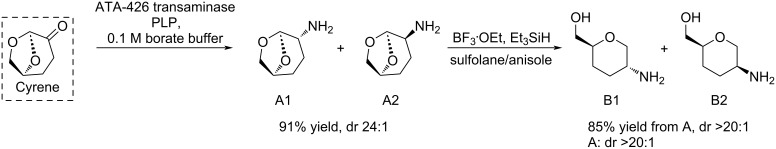
Synthesis of 5-amino-2-(hydroxymethyl)tetrahydropyran from Cyrene^®^.

Cyrene^®^ has also been used as a starting chiral building block for applications in drug design. For example, 5-amino-2-hydroxymethyltetrahydropyran (B1), an intermediate in the synthesis of bioactive compounds, was synthesized via a two-step, protecting-group-free, process [437]. Cyrene^®^ underwent an enzyme-catalyzed (ATA-426) transamination followed by reductive acetal opening. ATA-426 provided the chiral cyclohexylamine intermediates **A1** and **A2** with high selectivity (24:1) and 91% yield. Hydroxy-amino derivatives **B1** and **B2** were obtained from **A1** and **A2** in 85% yield with a high diastereoselectivity (>20:1). This two-step process replaced the reported nine-step synthesis of **B1** from tri-*O*-acetyl-ᴅ-glucal and resulted in a more than 27% yield improvement (Scheme 155).

## Conclusion

The valorization of biobased carbonyl compounds represents a pivotal strategy in the transition towards a greener and sustainable biobased chemical industry. The low-molecular-weight intermediates derived from renewable biomass resources exhibit versatile reactivity and serve as crucial building blocks for a wide array of value-added chemicals, fuels, and other materials. Significant advances in catalytic technologies, reaction engineering, and process integration have enabled efficient transformations of biobased aldehydes, ketones, and related compounds into higher-value products such as alcohols, acids, esters, polymers, and fine chemicals. The remarkable structural diversity offered by biobased carbonyl compounds, and the immense variety of chemical reactions that carbonyl compounds can undergo, create an extremely powerful opportunity for accessing all kinds of chemical architectures, including complex ones. Despite the recent progress in the field, efforts must be continued for tackling the remaining challenges, notably achieving always higher selectivity, catalytic stability, and process scalability under economically and environmentally favorable conditions. This will constitute the basis for future biobased access to all types of chemical products.

## List of Abbreviations

The abbreviations used in the text and schemes are collected in Table 1.

**Table 1:** List of abbreviations.

Name	Abbreviation

ALA	atrolactic acid
BA	benzylic acid
CMF	5-(chloromethyl)furfural
CMFCC	5-(chloromethyl)-furan-2-carbonyl chloride
DES	deep eutectic solvent
DFF	2,5-diformyfuran
DHA	dihydroxyacetone
DHM	dihydroxymethylfuran
DHMF	2,5-dihydroxymethylfurfural
DHMTHF	dihydroxymethyltetrahydrofuran,
DMF	2,5-dimethylfuran
DMTHF	dimethyltetrahydrofuran
EG	ethylene glycol
EL and BL	ethyl levulinate and butyl levulinate
FA	furfuryl alcohol
FDCA	furandicarboxylic
FDCC	furan-2,5-dicarbonyl chloride
FFCA	5-formyl-2-furancarboxylic acid
GA	glycolic acid
GBL	γ-butyrolactone
GCA	glycolaldehyde
GLAD	glyceraldehyde
GLY	glycerol
GVL	γ-valerolactone
H_2_LGO	dihydrolevoglucosenone
Hap	hydroxyapatite
HBO	(*S*)-γ-hydroxymethyl-α,β-butenolide
HCPN	3-(hydroxymethyl)cyclopentanone
HCPN	3-(hydroxymethyl)cyclopentanone
HDGK	2,5-hexadione glycerol ketal
HFO	2-hydroxy-2(5*H*)-furanone
2H-HBO	(*S*)-γ-hydroxymethyl butyrolactone
HHCPN	4-hydroxy-4-(hydroxymethyl)cyclopent-2-en-1-one
HHD	1-hydroxyhexane-2,5-dione
HMCP	2-hydroxy-3-methylcyclopent-2-enone
HMF	5-hydroxymethylfurfural
HMFA	5-hydroxymethylfuranoic acid
HMTHFF	hydroxymethyltetrahydrofurfural
2,3-HPO	2,3-dihydroxypropanal
3-HPO	3-hydroxypropanal
HXD	2,5-hexanedione
IB	α-hydroxyisobutyric acid
LA	lactic acid
LEV	levulinic acid
LGO	levoglucosenone
Lgol	levoglucosanol
LMW-H	low-molecular-weight humin
MA	mandelic acid
MAN	maleic anhydride
MCP	3-methylcyclopent-2-enone
2-MeTHF	2-methyltetrahydrofuran
MFA	methylfurfurylalcohol,
MS	molecular sieves
MSA	methane sulfonic acid
M-THP-2H-HBO	α-methylenated derivative
NADH	nicotinamide dinucleotide
PA	propanoic acid
PDO	1,3-propanediol
PGA	polyglycolic acid
PLA	polylactic acid
PLGA	polylactic-*co*-glycolic acid
PX	*p*-xylene
RAFT	reversible addition–fragmentation chain transfer
SGCN	mesoporous graphite carbon nitride
TBD	1,5,7-triazabicyclo[4.4.0]dec-5-ene
ThdP-lyase	thiamine diphosphate-dependent lyase
THFDM	tetrahydrofurandimethanol
THP	2-tetrahydropyranyl
TS-1	titanium silicalite catalyst
TsOH·H_2_O	*p*-toluenesulfonic acid monohydrate
U-4C-3CR	Ugi 4-center 3-component reaction
VA	valeric acid
VEs	valerate esters

## Data Availability

Data sharing is not applicable as no new data was generated or analyzed in this study.
